# Socio-economic inequality underpins inequity in influenza vaccination uptake between public and private secondary schools: an Australian population-based study

**DOI:** 10.1016/j.lanwpc.2025.101761

**Published:** 2025-11-28

**Authors:** Huong Le, Christopher C. Blyth, Clement Schlegel, Jo-Anne Morgan, Francis Mitrou, Ha Nguyen, Rachel Foong, Samantha Carlson, Catherine Hughes, Bette Liu, Hannah C. Moore

**Affiliations:** a Wesfarmers Centre of Vaccines and Infectious Diseases, The Kids Research Institute Australia, Nedlands, Perth, Australia; b School of Population Health, Curtin University, Perth, Australia; c Australian Government Department of Health and Aged Care, Canberra, Australia; d Communicable Disease Control Directorate, Western Australian Department of Health, Perth, Australia; e ARC Centre of Excellence for Children and Families over the Life Course, Australia; f Human Development and Community Wellbeing Team, The Kids Research Institute Australia, Nedlands, Perth, Australia; g Immunisation Foundation of Australia, Australia; h National Centre for Immunisation Research and Surveillance, Australia; i School of Population Health, University of New South Wales, Sydney, Australia; j Department of Microbiology, PathWest Laboratory Medicine, QEII Medical Centre, Nedlands, Perth, Australia; k Department of Infectious Diseases, Perth Children's Hospital, Nedlands, Perth, Australia; l School of Medicine, The University of Western Australia, Nedlands, Perth, Australia; m Centre for Child Health Research, The University of Western Australia, Nedlands, Perth, Australia

**Keywords:** Influenza vaccination uptake, Vaccination inequity, Socio-economic inequality, Children, Public versus private schools

## Abstract

**Background:**

Socio-economic inequality and vaccination inequity have long been critical issues. However, no studies have explored the gap in influenza vaccination uptake between public and private schools. Importantly, the extent to which socio-economic inequality translates into vaccination uptake inequity has not been quantified. We investigate influenza vaccination uptake among school-aged Australian children in 2023, compare uptake between public and private schools, and assess the role of socio-economic inequality in vaccination uptake inequity.

**Methods:**

We analysed whole-of-population linked immunisation, census, and administrative data. Multivariable logistic regression was used to identify key uptake predictors, and the Oaxaca-Blinder decomposition was used to identify factors driving uptake inequity between public and private schools.

**Findings:**

Of 9.5 million influenza vaccination doses administered, only 0.7 million (7%) were given to school-aged children (5–<18 years), who represent 16% of the population. Coverage among school-aged children was low. Secondary school-aged children had the lowest uptake, with a significant gap between public and private schools. Children in private secondary schools, who demonstrate greater socio-economic advantage, had higher uptake than their public peers (unadjusted OR = 1.47; 95% CI: 1.45–1.57). Two-thirds of the uptake gap is driven by differences in cultural, linguistic, and socio-economic characteristics, with parental education, parental income, and socio-economic characteristics of residential area being the strongest contributors.

**Interpretation:**

Addressing socio-economic inequality among parents could reduce vaccination uptake inequity for children. Future influenza vaccination campaigns should consider tailored strategies for specific cultural, linguistic, and socio-economic groups.

**Funding:**

Wesfarmers Centre of Vaccines and Infectious Diseases; Western Australian’s Future Health Research and Innovation Fund.

**Research in context**

**Evidence before this study**

We searched PubMed, Embase, CINAHL, and Scopus databases for articles published between 2000 and 2025 using the terms “socio-economic inequality” AND “influenza vaccination uptake” AND “children” AND “public private schools”. No study on the issue was found. While existing studies have shown that socio-economic characteristics are important predictors for children's vaccination uptake, none of the studies have explored the influenza vaccination uptake gap between children attending public and private schools. Importantly, the extent to which inequality in socio-economic characteristics between children attending the two school types translates into inequity in vaccination uptake has not been quantified.

**Added value of this study**

This is the first study to uncover disparity in influenza vaccination uptake between children attending public and private schools and apply a robust statistical decomposition method to pinpoint the absolute and relative importance of factors behind the vaccination uptake inequity. By analysing whole-of-population Australian data and leveraging novel features of the Australian Immunisation Register system, which accurately records all vaccines administered to the population, we overcome possible limitations in previous studies. These include biases due to under-reporting of influenza vaccines, sample selection, and population non-representativeness in survey data. Additionally, by utilising the unique features of the Australian Census, we are able to link children with their parents (mothers and fathers separately). As a result, for the first time in the literature, we capture a rich set of health, cultural, linguistic, socio-economic, and demographic characteristics of both children and their parents.

We show that of the 9.5 million influenza vaccination doses administered in Australia in 2023, only 0.7 million (or 7%) were given to school-aged children (those 5 to <18 years old), despite this group making up 16% of the population. Coverage in school-aged children was low. Children in private secondary schools, who demonstrate greater socio-economic advantage, had a notable higher vaccination uptake than their public peers (unadjusted OR = 1.47; 95% CI: 1.45–1.57). Approximately two-thirds of the uptake gap is explained by differences in cultural, linguistic, and socio-economic characteristics. The largest contributors come from parental education, parental income, and area-level socio-economic characteristics.

**Implications of all the available evidence**

Our results show that socio-economic inequality plays an important role in shaping vaccination uptake inequity. Therefore, addressing socio-economic inequality among parents is expected to reduce vaccination uptake inequity for children. Furthermore, given a strong association exists between socio-economic, cultural and linguistic factors and children's vaccination uptake, future influenza vaccination campaigns should consider tailored strategies for specific cultural, linguistic, and socio-economic groups. Even with the higher influenza vaccination coverage among Australian private school children (of 19.6% in 2023) than public school peers, coverage remains low. Alternative methods of seasonal influenza vaccination delivery for school-aged Australian children, such as the live attenuated influenza vaccine in a nasal spray and a school-based program, to improve uptake could be explored.

## Introduction

Influenza and its complications are an important global cause of hospitalisations and deaths. In Australia, children suffer a disproportionate burden. Children aged 0–<18 years represent 21% of the population, but accounted for 48% of all national influenza notifications and more than 72% of influenza hospitalisations.^1^, ^2^, ^3^, ^4^ Of all influenza notifications in children, 75% occurred in school-aged children (5–<18 years old).^3^

Seasonal influenza vaccine (SIV) is the most effective tool to prevent severe influenza infection.^5^, ^6^, ^7^ In addition to limiting the infection, influenza vaccination can also reduce hospitalisations from other pathogens like *Streptococcus pneumoniae* and respiratory syncytial virus.^8^^,^^9^ Our recent modelling study for Western Australia (WA) shows that vaccinating school-aged children effectively reduces transmission, protects not just children but the broader community, and saves millions of public health costs.^10^

SIV has been recommended and funded for all pre-school aged children (those 6 months to <5 years) in WA since 2008. This funding for pre-school-aged children has been expanded nation-wide under the National Immunisation Program (NIP) since 2020.^11^ WA has funded SIV for primary school-aged children (5–11 years) since 2020 and all WA residents during the months of SIV campaigns since 2022. Queensland has also funded SIV for all Queenslanders since 2022 (May–Jul 2022; extending to May–Aug 2023, and April–Sep 2024) following a significant influenza outbreak with high rates of unvaccinated patients and influenza associated deaths.^12^^,^^13^ Despite these funded programs, influenza vaccination coverage among children remains low.^14^ Notably, coverage in secondary school-aged children is lowest in the population with concerning declines relative to pandemic peaks.^14^, ^15^, ^16^ To enhance protection against influenza and close the widening immunity gaps in the population by age groups, it is important to understand the underlying reasons for the low uptake.

Socio-economic inequality and vaccination uptake inequity^n^ have long been critical public health concerns. Existing studies have shown that vaccination uptake varies by socio-economic status, race, ethnicity, and religion that individuals belong to.^17^, ^18^, ^19^, ^20^, ^21^, ^22^ To our knowledge, none of the studies have explored the uptake gap among children by school-type. Importantly, the extent to which inequality in socio-economic characteristics translates into inequity in influenza vaccination uptake has not been quantified.

Australia is a unique setting for this study, as socio-economic disparity between children attending public and private schools in Australia is among the highest worldwide and the second highest among OECD countries.^23^^,^^24^ Disparity in socio-economic characteristics, which are key predictors for vaccination uptake,^17^ is expected to result in a discrepancy in influenza vaccination uptake. This leads to unequal opportunities for children in different school type to be protected against serious consequences of influenza infection; to maintain regular school attendance and participate in other developmental activities. Furthermore, social differences in vaccination uptake can prevent herd immunity, thereby increasing the risk of localised influenza outbreaks.^18^ Such differences may exacerbate the existing social divide. Understanding social differences in vaccination uptake by school type can inform targeted public health interventions to promote vaccine equity and protect vulnerable populations.

Utilising the novel whole-of-population Australian Immunisation Register linked with the 2021 Australian Census and other administrative data, we investigate influenza vaccination uptake among school-aged children in 2023. We compare uptake across public and private schools, identify important uptake predictors, and quantify the extent to which socio-economic inequality translate into inequity in influenza vaccination uptake between public and private secondary schools. Our study provides new insights into how structural barriers shape vaccination uptake inequity.

## Methods

### Study design and data source

We undertook a population-based cohort study using data from the nation-wide whole-of-population Australian Immunisation Register (AIR) linked with Medicare, Core Demographic, Core Indigenous, and the 2021 Census of Population and Housing data. All influenza vaccine doses administered in Australia during 2023 were extracted from the AIR, and the active Australian population was identified from the Medicare database. Information on children's school type, socio-economic, demographic, health as well as characteristics of their parents and residential areas, was obtained through the Census, Core Demographic, and Core Indigenous data.

Datasets were linked via a Person Linkage Spine, an individual identification key created by the Australian Bureau of Statistics (ABS). This Linkage Spine was deterministically generated based on individuals’ first name, last name, address, date of birth, and gender.^25^ We used version six of the Spine, which broadly covers all residential population in Australia from 2006 onwards.^25^ We accessed and analysed the de-identified data remotely via a secure virtual machine administered by the ABS. All our analytical outputs were vetted by the ABS.

Below we describe in detail each data set.

• Medicare Registration database

Medicare is Australia's universal public health insurance scheme. All Australian citizens, permanent residents, New Zealand citizens living in Australia, and in certain circumstances oversea visitors applying for permanent residence visa, have a Medicare card.^26^ People with Medicare have access to a wide range of government-subsidised health care services at low or no cost. Approximately 99% of all children residing in Australia are covered by Medicare.^27^

• Australian Immunisation Register

The Australian Immunisation Register (AIR), expanded from the Australian Childhood Immunisation Register (ACIR), records vaccination data for all Australians, from birth to death, since 2016.^16^ AIR includes detailed vaccination information such as brand/type, date of receipt, dose number, claim number, and provider. Following the Government's amendment to the AIR Reporting Act, from 1 March 2021, all influenza vaccines administered to the population is mandatory to report in the AIR.^28^

• 2021 Australian Census of Population and Housing

The Australian Census of Population and Housing, herein referred as the Census is conducted every five years. It collects key cultural, social, economic, and demographic information from all people residing in Australia on Census night, with the sole exception of foreign diplomats.

The 2021 Census, held on 10 August 2021, was the 18th and most recent to date. It provides information on individuals, families, and dwellings. At the individual level, it has information on gender, date of birth, ancestry, country of birth, Aboriginal and/or Torres Strait Islander (herein referred to as Aboriginal) status, English proficiency, additional languages spoken at home, religion, marital status, education, employment, income, occupation, and skill level of occupation. Unlike previous Censuses, the 2021 Census additionally collected information on long-term health conditions classified into one of ten major diseases including arthritis, asthma, cancer, dementia, diabetes, heart disease, kidney disease, lung conditions, mental health conditions, stroke, and other long-term health conditions.

At the family and dwelling level, it provides information on family type (i.e., single or couple-parent family) and composition, dwelling type, size, structure, and location. Using recorded residential locations, the ABS produced variables indicating Accessibility/Remoteness for areas of residence and relative Socio-Economic Indexes for Area (SEIFA). The Accessibility/Remoteness variable is derived by measuring road distance to nearest service centres using population as a proxy measure for service availability, classified into one of five categories: ‘Major cities’, ‘Inner regional’, ‘Outer regional’, ‘Remote’, and ‘Very remote’.^29^ Of SEIFA variables, we use the Index of Relative Socio-economic Advantage and Disadvantage (IRSAD) measured at Statistical Area Level 1 and ranked by deciles.

In each family (household), the Census has an indicator for the family reference person, spouse or partner of family reference person, and relationships of other household members to the family reference person. This allows us to identify mothers and fathers within each family, and link children to their parents. Therefore, for the first time in the literature, we are able to control for a rich set of variables observed for mothers and fathers separately.

Importantly, the Census collected information on the type of educational institution a person was attending. For those in primary and secondary schools, an additional question about school type, categorised as Government (Public), Non-Government (Private), or Catholic, was included. This unique feature makes our study possible.

• Core Demographic and Core Indigenous data

The Core Demographic and Core Indigenous data, produced by the ABS, validate information from various administrative sources and censuses. Administrative datasets used include the Medicare Consumer Directory, Centrelink Administrative Records, Australian Tax Office Client Register, and Birth and Death Registrations. These core datasets provide accurate information on individual demographic characteristics, and Aboriginal status for all Australians.

### Population and outcome of interest

When exploring influenza vaccine coverage rates in 2023, our population of interest is the entire population, and subgroups classified by age and jurisdiction.

Since secondary school-aged children have the lowest influenza vaccination coverage and the largest coverage gap between public and private schools, we focus on this group when identifying key predictors for influenza vaccination uptake and quantifying the relative importance of factors contributing to the uptake gap between public and private schools. Supplementary Material S1 provides further details on the secondary school-aged children identification.

Our *outcome* of interest is influenza vaccination status, coded as a binary variable indicating whether children received influenza vaccine in 2023 (the most recent year with available data at the time of analysis).

### Statistical analyses

#### Main analysis

First, we calculated influenza vaccination coverage for the entire population and subpopulation groups, broken down by age and jurisdictions in 2023. Next, we calculated coverage among school-aged children by education level and school type.

We then compared characteristics of those attending public and private secondary schools. Multivariable logistic regression model was used to identify important predictors for influenza vaccination uptake. To capture the entire population cohort, missing values in categorical variables were assigned to a separate category, and a binary indicator for this group was included in the regression model. Further details on observed variables and model specification for the logistic regression are in Supplementary Table S3 and Supplementary Material S1, respectively.

The Oaxaca-Blinder decomposition method, applied to logistic regression models, was employed to quantify the relative importance of factors contributing to the coverage gap between children attending public and private secondary schools. Specifically, it estimates how much of the gap is explained by inequality in socio-economic characteristics, and by disparity in health, cultural, linguistic, and demographic factors of children, their parents, families, and residential areas; and how much remains unexplained. This unexplained component is due to differences in the estimated coefficients or other factors not captured in the model.^30^ Further details on the Oaxaca-Blinder decomposition method are in Supplementary Material S1. All contributing factors are in absolute (and relative values) with 95% confidence intervals (CI).

#### Robustness check

In main analysis, we classified children into school types using information in the Census. For children who first enrolled Year 1 in 2022 or 2023, information about their school-type was unavailable. Therefore, we restricted our analysis to children ≥ 8 years old when calculating influenza vaccination coverage. There is a possibility that we might not capture correct school type for children who changed school type between the 2021 Census and 2023, and school level for those who repeated a grade. Given the low likelihood of these events, we expect this did not affect our results. As a robustness check, we conducted similar analyses using influenza vaccine administered in 2021, the same year as the Census, for validation.

### Role of the funding source

This study was funded by the Wesfarmers Centre of Vaccines and Infectious Diseases' Seed Grant, and the Western Australian's Future Health Research and Innovation Fund (Grant ID WANMAIdeas2025-25/7). The funders had no role in the design, analysis, interpretation, or writing of the manuscript.

### Ethics approval

This project used secondary, de-identified person level data produced from the Australian Government's Multi-Agency Data Integration Project (now known as PLIDA: Population Level Integrated Data Asset). We remotely accessed and analysed the data via a secure virtual machine administered by the ABS. All analytical outputs were approved by the ABS before releasing. This project does not involve any human or animal experiments. Human Research Ethics Exemption was approved by the University of Western Australia's Human Research Ethics Committee.

## Results

### Influenza vaccination uptake in children attending public and private secondary schools

**Fig. 1:**
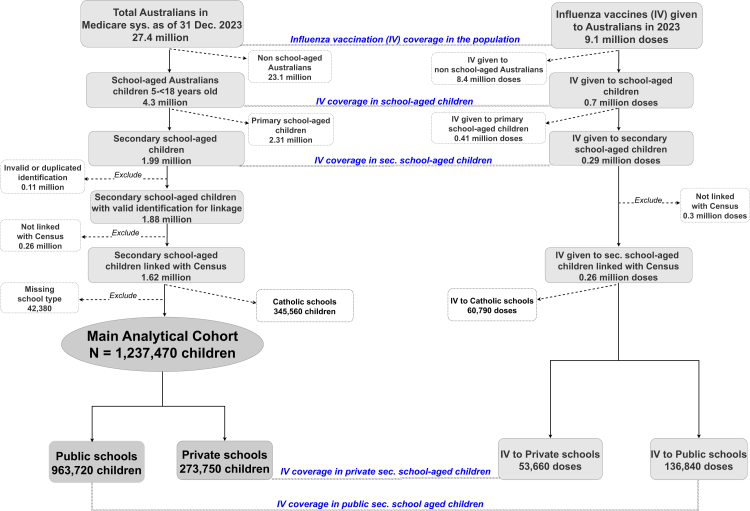
Flowchart of data used.

Fig. 1 presents a flow chart of the data used. There were 27.4 million Australians active in the Medicare system in 2023. Of them, 4.3 million (16%) were school-aged children. Within this group, 1.99 million were secondary school-aged, with 963,720 attending public and 273,750 attending private schools (Fig. 1).

**Fig. 2:**
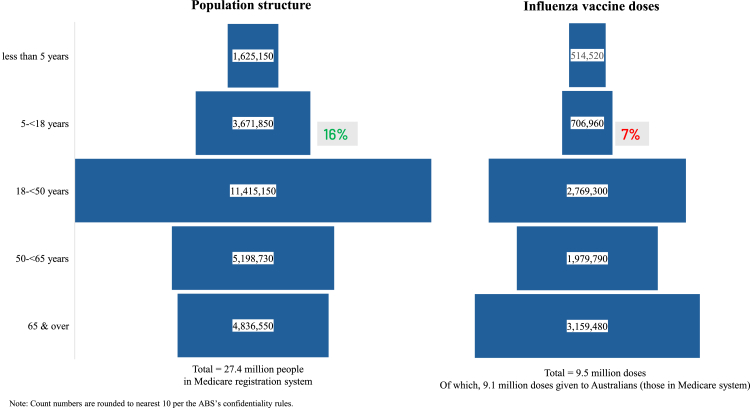
Population structure and influenza vaccination doses administered by age groups in 2023. Note: Count numbers are rounded to nearest 10 per the ABS's confidentiality rules.

A total of 9.5 million influenza vaccine doses were administered across the population in 2023. Of these, 9.1 million (or 96%) were given to Australians (those in the Medicare system) and 0.4 million (or 4%) to non-Australians (such as visitors or temporarily migrants). Of all doses administered, only 0.7 million (7%) were given to school-aged children, who comprise 16% of the population (Fig. 2).

**Fig. 3:**
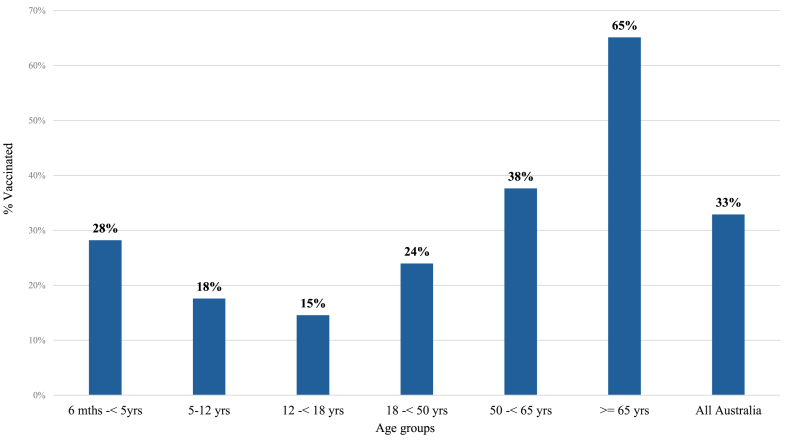
Influenza vaccination coverage by age-groups in 2023.

In 2023, influenza vaccination uptake in school-aged children was the lowest of all age groups. Coverage was 15% for secondary, and 18% for primary school-aged children nationwide. This was well below coverage of 33% in overall population, and coverage of 28% for pre-school-aged children, 24% for adults aged 18 to <50 years, 38% for adults aged 50 to <65 years, and 65% for the elderly (Fig. 3).

Low influenza vaccination coverage among school-aged children was consistently observed across all jurisdictions. While coverage is highest in the Australian Capital Territory, coverage in Queensland is among the lowest (Supplementary Table S1).

**Fig. 4:**
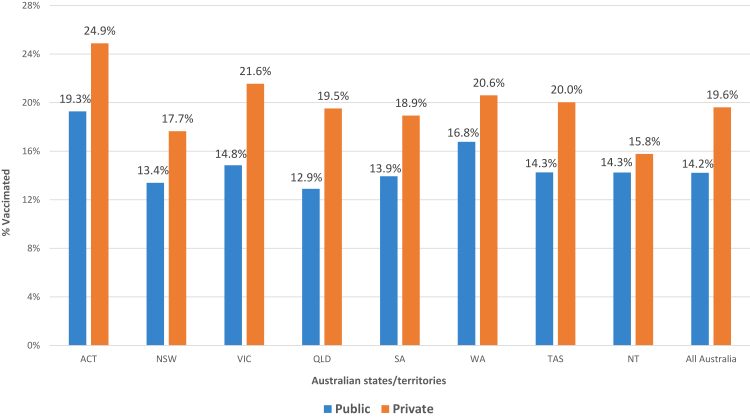
Influenza vaccination coverage in children attending public vs. private secondary schools by jurisdictions across Australia in 2023.

There is a significant gap in coverage between children in public and private schools, and the gap is wider for higher levels of education. At the secondary education level, coverage is only 14.2% in public schools, but 19.6% in private schools. The unadjusted odds ratio (OR) of coverage between private vs. public secondary schools is 1.47 (95% CI: 1.45–1.57). Higher coverage in private than public schools consistently occurs in all states and territories. The coverage gap between public and private schools is highest in Victoria and Queensland and lowest in Northern Territory, where children of both school types have low coverage (Fig. 4 & Supplementary Table S2).

### Comparative characteristics for those attending public and private secondary schools

Public schools have a higher proportion of children and parents who are non-Australian citizens, Aboriginal and/or Torres Strait Islander, without a religion, or with long-term health conditions. Children in private schools have older and more educated parents with advanced occupational skills holding managerial or professional positions, private sector employment, and higher income. For example, 53% of mothers and 42% of fathers of children in private schools hold a bachelor's degree or higher, whereas the rate is only 30% for mothers and 21% for fathers of children in public schools. Similarly, 42% of mothers and 48% of fathers of children in private schools are managers or professionals, compared with only 25% of both parents of children in public schools. More parents of children in public schools are labourers, trade workers or clerical employees than those in private schools (Supplementary Table S3).

A higher proportion of parents of children in private schools work full-time. Private school mothers work an average of 23 h per week versus 20 h for public school mothers–a 15% difference. The gap is more pronounced for fathers, who work 31.5 versus 25 h per week in private versus public schools -a 26% difference. Parents of children in private schools have higher income: 44% of fathers and 23% of mothers earn ≥ AUD $104.000 per year, compared with only 23% of fathers and 9% of mothers in public schools.

A higher proportion of children in private schools come from families in which mothers and fathers are legally married. More than 25% of children in public schools come from single-parent families, while the rate is less than 14% for children in private schools. Furthermore, a higher proportion of children in private schools live in advantaged socio-economic areas (Supplementary Table S3

### Predictors of influenza vaccination uptake in children attending public and private secondary schools

Results from the logistic regressions, identifying important predictors for influenza vaccination uptake in secondary school-aged children by school types, are presented in Table 1. Girls are more likely to be vaccinated than boys. Children with Australian citizenship are more likely to be vaccinated. Speaking an additional language at home is strongly associated with influenza vaccination uptake. Children who speak Eastern European, Southern European, Southwest Asian, or Central Asian languages as an additional language at home are less likely to be vaccinated than those who speak English only or Northern European languages (the reference group). In contrast, children who speak Southeast Asian or Eastern Asian languages at home are more likely to be vaccinated than the reference group.

**Table 1:** Predictors of influenza vaccination uptake in secondary school-aged children by school-types - result from logistic regressions.

Variables	School types			
	Public		Private	
	Model 1	Model 2	Model 1	Model 2
	Odds ratio [SE]	Odds ratio [SE]	Odds ratio [SE]	Odds ratio [SE]
*Child characteristics:*				
Age^a^^‽^	0.977∗∗∗ [0.002]	0.979∗∗∗ [0.002]	1.018∗∗∗ [0.002]	1.019∗∗∗ [0.002]
Aboriginal and/or Torres Strait Islander				
No	Ref. group	Ref. group	Ref. group	Ref. group
Yes	1.219∗∗∗ [0.015]	1.122∗∗∗ [0.018]	0.991 [0.033]	0.958 [0.037]
NA∗∗ or missing	0.550∗∗∗ [0.028]	0.529∗∗∗ [0.030]	0.570∗∗∗ [0.046]	0.567∗∗∗ [0.049]
Gender				
Boy	Ref. group	Ref. group	Ref. group	Ref. group
Girl	1.126∗∗∗ [0.007]	1.111∗∗∗ [0.007]	1.107∗∗∗ [0.011]	1.106∗∗∗ [0.012]
Australian citizenship				
Yes	Ref. group	Ref. group	Ref. group	Ref. group
No	0.822∗∗∗ [0.011]	0.832∗∗∗ [0.012]	0.877∗∗∗ [0.024]	0.865∗∗∗ [0.026]
NA (not available) or missing	0.804∗∗∗ [0.047]	0.758∗∗∗ [0.053]	0.920 [0.092]	0.928 [0.101]
Long-term health condition(s)				
None	Ref. group	Ref. group	Ref. group	Ref. group
≥1 long-term health condition	1.654∗∗∗ [0.012]	1.625∗∗∗ [0.014]	1.480∗∗∗ [0.019]	1.458∗∗∗ [0.020]
NA or missing	1.044∗∗∗ [0.016]	1.045∗∗ [0.019]	1.046 [0.029]	1.060∗ [0.032]
Additional language spoken at home				
Northern Europe including English	Ref. group	Ref. group	Ref. group	Ref. group
Southern Europe	0.658∗∗∗ [0.020]	0.652∗∗∗ [0.021]	0.574∗∗∗ [0.027]	0.572∗∗∗ [0.029]
Eastern Europe	0.304∗∗∗ [0.014]	0.288∗∗∗ [0.015]	0.374∗∗∗ [0.031]	0.396∗∗∗ [0.034]
Southwest & Central Asian	0.706∗∗∗ [0.018]	0.714∗∗∗ [0.020]	0.592∗∗∗ [0.027]	0.610∗∗∗ [0.030]
Southern Asian	1.126∗∗∗ [0.023]	1.068∗∗∗ [0.023]	1.134∗∗∗ [0.040]	1.102∗∗∗ [0.040]
Southeast Asian	1.775∗∗∗ [0.034]	1.780∗∗∗ [0.037]	1.770∗∗∗ [0.080]	1.761∗∗∗ [0.085]
Eastern Asian	1.341∗∗∗ [0.024]	1.312∗∗∗ [0.026]	1.414∗∗∗ [0.041]	1.448∗∗∗ [0.045]
Aus. Aboriginal Languages	1.629∗∗∗ [0.076]	1.877∗∗∗ [0.109]	1.487∗∗∗ [0.178]	1.493∗∗∗ [0.203]
Other languages	0.618∗∗∗ [0.022]	0.595∗∗∗ [0.025]	0.650∗∗∗ [0.045]	0.658∗∗∗ [0.050]
NA or missing	0.959 [0.033]	0.982 [0.040]	0.918 [0.067]	0.915 [0.074]
*Mother's characteristics:*				
English proficiency				
Speaks English only	Ref. group	Ref. group	Ref. group	Ref. group
English very well & use other language	1.038∗∗∗ [0.013]	1.037∗∗∗ [0.015]	0.934∗∗∗ [0.019]	0.932∗∗∗ [0.020]
English not well/not at all & other languages	1.347∗∗∗ [0.029]	1.366∗∗∗ [0.033]	1.182∗∗∗ [0.054]	1.225∗∗∗ [0.063]
Religion				
Christian	Ref. group	Ref. group	Ref. group	Ref. group
Buddhism	1.196∗∗∗ [0.020]	1.215∗∗∗ [0.022]	1.233∗∗∗ [0.043]	1.287∗∗∗ [0.048]
Hinduism	0.856∗∗∗ [0.019]	0.834∗∗∗ [0.020]	1.031 [0.041]	0.995 [0.041]
Islam	0.752∗∗∗ [0.015]	0.772∗∗∗ [0.016]	0.607∗∗∗ [0.021]	0.618∗∗∗ [0.022]
Judaism	1.171∗∗∗ [0.060]	1.125∗∗ [0.062]	1.399∗∗∗ [0.048]	1.378∗∗∗ [0.049]
Aboriginal cultural beliefs or other religions	0.605∗∗∗ [0.017]	0.622∗∗∗ [0.019]	0.626∗∗∗ [0.037]	0.664∗∗∗ [0.041]
No religion or secular belief	0.881∗∗∗ [0.006]	0.892∗∗∗ [0.007]	0.980∗ [0.011]	0.997 [0.012]
NA or missing	0.747∗∗∗ [0.018]	0.747∗∗∗ [0.022]	0.835∗∗∗ [0.035]	0.852∗∗∗ [0.040]
Highest education obtained				
Post-graduate	Ref. group	Ref. group	Ref. group	Ref. group
Graduate diploma & graduate certificate	1.011 [0.017]	1.015 [0.018]	0.915∗∗∗ [0.021]	0.927∗∗∗ [0.022]
Bachelor degree	0.957∗∗∗ [0.010]	0.966∗∗∗ [0.011]	0.885∗∗∗ [0.013]	0.904∗∗∗ [0.014]
Advanced diploma & diploma	0.704∗∗∗ [0.009]	0.772∗∗∗ [0.011]	0.644∗∗∗ [0.013]	0.709∗∗∗ [0.015]
Certificate I to IV levels	0.663∗∗∗ [0.008]	0.744∗∗∗ [0.011]	0.616∗∗∗ [0.013]	0.691∗∗∗ [0.016]
Years10–12	0.637∗∗∗ [0.008]	0.721∗∗∗ [0.010]	0.574∗∗∗ [0.012]	0.644∗∗∗ [0.014]
Years 9 & below	0.732∗∗∗ [0.015]	0.838∗∗∗ [0.021]	0.688∗∗∗ [0.042]	0.799∗∗∗ [0.058]
NA or missing	1.153∗∗∗ [0.038]	1.343∗∗∗ [0.059]	0.926 [0.100]	0.949 [0.130]
Hours worked in all jobs in the week prior to census	0.998∗∗∗ [0.000]	0.998∗∗∗ [0.000]	0.996∗∗∗ [0.000]	0.997∗∗∗ [0.000]
Total personal income				
<$64.999/year	Ref. group	Ref. group	Ref. group	Ref. group
$65.000–$103.999/year	1.055∗∗∗ [0.009]	1.039∗∗∗ [0.010]	1.099∗∗∗ [0.016]	1.085∗∗∗ [0.017]
≥104.000/year	1.168∗∗∗ [0.013]	1.112∗∗∗ [0.014]	1.295∗∗∗ [0.019]	1.228∗∗∗ [0.020]
NA or missing	0.826∗∗∗ [0.022]	0.919∗∗ [0.033]	0.771∗∗∗ [0.037]	0.907∗ [0.053]
Long-term health condition(s)				
None	Ref. group	Ref. group	Ref. group	Ref. group
≥1 long-term health cond.	1.175∗∗∗ [0.008]	1.137∗∗∗ [0.009]	1.238∗∗∗ [0.014]	1.219∗∗∗ [0.015]
NA or missing	1.021 [0.018]	1.023 [0.021]	1.048 [0.031]	1.044 [0.034]
*Father's characteristics:*				
Highest education obtained				
Post-graduate		Ref. group		Ref. group
Graduate diploma & graduate certificate		1.047∗∗ [0.023]		0.908∗∗∗ [0.025]
Bachelor degree		0.996 [0.012]		0.939∗∗∗ [0.014]
Advanced diploma & diploma		0.857∗∗∗ [0.013]		0.766∗∗∗ [0.017]
Certificate I to IV level		0.738∗∗∗ [0.010]		0.675∗∗∗ [0.014]
Years10–12		0.763∗∗∗ [0.011]		0.751∗∗∗ [0.017]
Years 9 & below		0.826∗∗∗ [0.019]		0.751∗∗∗ [0.047]
NA or missing		0.869∗∗∗ [0.041]		1.073 [0.155]
Skill level of occupation				
Level 1 (highest skill)		Ref. group		Ref. group
Level 2		0.939∗∗∗ [0.012]		0.932∗∗∗ [0.018]
Level 3		0.856∗∗∗ [0.011]		0.887∗∗∗ [0.020]
Level 4		0.883∗∗∗ [0.010]		0.972 [0.022]
Level 5 (lowest skill)		0.884∗∗∗ [0.014]		0.920∗∗ [0.030]
NA or missing		0.879∗∗∗ [0.014]		1.003 [0.026]
Hours worked in the week prior to census		1.001∗∗∗ [0.000]		1.001∗∗∗ [0.000]
Total personal income				
≤ $64.999/year		Ref. group		Ref. group
$65.000–$103.999/year		1.018∗ [0.010]		1.023 [0.020]
≥104.000/year		1.125∗∗∗ [0.012]		1.217∗∗∗ [0.023]
NA or missing		0.826∗∗∗		0.936
Long-term health condition(s)				
None		Ref. group		Ref. group
≥1 long-term health cond.		1.186∗∗∗ [0.009]		1.168∗∗∗ [0.015]
NA or missing		0.962∗∗ [0.019]		0.942∗ [0.030]
*Household & family characteristics:*				
Family type				
Couple parents	Ref. group		Ref. group	
Single parents	0.678∗∗∗ [0.006]		0.701∗∗∗ [0.012]	
Household size	0.911∗∗∗ [0.003]	0.910∗∗∗ [0.003]	0.891∗∗∗ [0.004]	0.887∗∗∗ [0.005]
Country of birth of parents				
Both parents born in Australia	Ref. group	Ref. group	Ref. group	Ref. group
One parent born oversea	0.975∗∗∗ [0.008]	0.948∗∗∗ [0.009]	0.950∗∗∗ [0.012]	0.925∗∗∗ [0.013]
Both parents born oversea	0.962∗∗∗ [0.011]	0.919∗∗∗ [0.011]	0.893∗∗∗ [0.015]	0.857∗∗∗ [0.015]
NA or missing	0.969 [0.042]	0.882∗ [0.058]	0.821∗ [0.085]	0.812 [0.106]
*Geographical &socio-economiccharacteristics of local area:*				
Remoteness				
Major cities	Ref. group	Ref. group	Ref. group	Ref. group
Inner Regional	0.963∗∗∗ [0.009]	0.973∗∗∗ [0.010]	1.043∗∗∗ [0.016]	1.065∗∗∗ [0.017]
Outer Regional	0.944∗∗∗ [0.012]	0.959∗∗∗ [0.014]	0.994 [0.026]	1.039 [0.029]
Remote	1.006 [0.028]	1.019 [0.032]	1.183∗∗ [0.085]	1.297∗∗∗ [0.098]
Very Remote	1.293∗∗∗ [0.049]	1.256∗∗∗ [0.055]	1.049 [0.125]	1.013 [0.134]
SEIFA of local living area				
0–10% (least advantage)	Ref. group	Ref. group	Ref. group	Ref. group
11–20%	1.007 [0.015]	1.015 [0.018]	0.985 [0.041]	0.974 [0.047]
21–30%	1.031∗∗ [0.015]	1.019 [0.018]	1.044 [0.041]	1.014 [0.046]
31–40%	1.001 [0.015]	0.989 [0.018]	1.078∗∗ [0.041]	1.025 [0.046]
41–50%	1.051∗∗∗ [0.015]	1.032∗ [0.018]	1.157∗∗∗ [0.043]	1.102∗∗ [0.048]
51–60%	1.131∗∗∗ [0.016]	1.100∗∗∗ [0.019]	1.279∗∗∗ [0.046]	1.194∗∗∗ [0.051]
61–70%	1.202∗∗∗ [0.017]	1.147∗∗∗ [0.020]	1.333∗∗∗ [0.048]	1.201∗∗∗ [0.051]
71–80%	1.323∗∗∗ [0.019]	1.232∗∗∗ [0.022]	1.468∗∗∗ [0.052]	1.299∗∗∗ [0.054]
81–90%	1.469∗∗∗ [0.022]	1.311∗∗∗ [0.024]	1.743∗∗∗ [0.061]	1.494∗∗∗ [0.062]
91–100% (most advantage)	1.642∗∗∗ [0.025]	1.402∗∗∗ [0.026]	2.155∗∗∗ [0.076]	1.781∗∗∗ [0.074]
SEIFA missing	0.978 [0.028]	1.099∗∗ [0.048]	2.323∗∗∗ [0.123]	2.071∗∗∗ [0.154]
State of residence				
NSW	Ref. group	Ref. group	Ref. group	Ref. group
VIC	1.193∗∗∗ [0.010]	1.183∗∗∗ [0.011]	1.326∗∗∗ [0.018]	1.294∗∗∗ [0.019]
QLD	1.105∗∗∗ [0.010]	1.115∗∗∗ [0.012]	1.245∗∗∗ [0.019]	1.243∗∗∗ [0.020]
SA	1.243∗∗∗ [0.017]	1.243∗∗∗ [0.020]	1.294∗∗∗ [0.028]	1.295∗∗∗ [0.030]
WA	1.419∗∗∗ [0.014]	1.403∗∗∗ [0.016]	1.399∗∗∗ [0.024]	1.390∗∗∗ [0.026]
TAS	1.364∗∗∗ [0.029]	1.389∗∗∗ [0.034]	1.320∗∗∗ [0.050]	1.231∗∗∗ [0.051]
NT	1.071∗∗ [0.037]	1.020 [0.041]	0.881∗ [0.058]	0.853∗∗ [0.060]
ACT	1.269∗∗∗ [0.026]	1.212∗∗∗ [0.028]	1.167∗∗∗ [0.043]	1.105∗∗ [0.043]
Constant	0.318∗∗∗ [0.011]	0.365∗∗∗ [0.015]	0.199∗∗∗ [0.013]	0.212∗∗∗ [0.016]
No. Observations	963,720	719,620	273,750	236,120

Note: Odds ratios are reported.

Standard errors (SE) are in square brackets.

Significant levels denoted by ∗∗∗ for P value <0.01, ∗∗ for P value <0.05, and ∗ for P value <0.1.

Number of observations are rounded to nearest 10 per the ABS's confidentiality rules.

Model 1 controls for child, mother, family and residential area's characteristics.

Model 2 adds to Model 1 father's characteristics, therefore applied to children in couple parents' families only.

a Age as of 30 June 2023.

Mothers' religion is strongly related to children's vaccination uptake. Children whose mothers reported their religion as Islamic, Australian Aboriginal and/or Torres Strait Islander cultural beliefs, or no religion are less likely to be vaccinated than those whose mothers are Christian (the reference group). Furthermore, long-term health conditions are strong predictors for vaccination uptake. Children, or those whose parents have one or more long-term health conditions, have higher odds of being vaccinated.

Parental education, skill level of occupation, and income are also strong predictors of children's vaccination uptake. Specifically, children of parents with higher education, more advanced occupational skill, and higher income are more likely to be vaccinated. The chance for children being vaccinated reduces slightly when mother works longer hours, but increases when father works longer hours.

Children from single-parent families are less likely to be vaccinated. Children living in areas of higher socio-economic advantage are more likely to be vaccinated. These patterns hold true for children in all school types (Table 1).

### Factors behind inequity in influenza vaccination coverage between public and private secondary schools

Table 2 presents result from the Oaxaca-Blinder decomposition applied to logistic regression model to quantify the relative importance of factors contributing to the uptake gap among children attending public vs. private secondary schools.

**Table 2:** Contributions to influenza vaccination coverage gap between children attending public vs. private secondary schools.

	Model 1		Model 2	
	Est. gap [relative contribution]	95% CI for est. gap	Est. gap [relative contribution]	95% CI for est. gap
**Panel A. Influenza Vaccination Coverage rates**				
Public schools	0.142∗∗∗	(0.141; 0.143)	0.153∗∗∗	(0.152; 0.154)
Private schools	0.196∗∗∗	(0.195; 0.198)	0.203∗∗∗	(0.201; 0.205)
**Panel B. Overall difference**	−0.054∗∗∗[100.0%]	(−0.056; −0.052)	−0.050∗∗∗ [100.0%]	(−0.052; −0.049)
Of which:				
Explained	−0.028∗∗∗ [51.1%]	(−0.028; −0.027)	−0.032∗∗∗ [63.3%]	(−0.033; −0.031)
Unexplained	−0.026∗∗∗ [48.9%]	(−0.028; −0.025)	−0.018∗∗∗ [36.7%]	(−0.021; −0.016)
**Panel C. Explained part - detail**				
*Child characteristics:*				
Age	0.000∗∗∗ [−0.8%]	(0.000; 0.001)	0.000∗∗∗ [−0.7%]	(0.000; 0.001)
Gender	0.000∗∗∗ [0.5%]	(0.000; 0.000)	0.000∗∗∗ [0.6%]	(0.000; 0.000)
Aboriginal/Torres Strait Islander indicator	0.001∗∗∗ [−1.9%]	(0.001; 0.001)	0.000∗∗∗ [−0.7%]	(0.000; 0.000)
Citizenship status	0.000∗∗∗ [0.9%]	(−0.001; 0.000)	−0.001∗∗∗ [1.4%]	(−0.001; −0.001)
Long-term health condition(s)	0.001∗∗∗ [−2.6%]	(0.001; 0.002)	0.001∗∗∗ [−1.7%]	(0.001; 0.001)
Additional language used at home	0.001∗∗∗ [−2.0%]	(0.001; 0.001)	0.001∗∗∗ [−2.4%]	(0.001; 0.001)
*Mother characteristics:*				
Religion	−0.001∗∗∗ [2.6%]	(−0.002; −0.001)	−0.001∗∗∗ [2.0%]	(−0.001; −0.001)
English proficiency	0.001∗∗∗ [−1.6%]	(0.001; 0.001)	0.001∗∗∗ [−2.6%]	(0.001; 0.001)
Highest education obtained	−0.012∗∗∗ [22.3%]	(−0.012; −0.012)	−0.009∗∗∗ [17.6%]	(−0.009; −0.008)
Hours worked the week prior to census	0.001∗∗∗ [−2.0%]	(0.001; 0.001)	0.001∗∗∗ [−1.5%]	(0.001; 0.001)
Total personal income from all jobs	−0.003∗∗∗ [6.2%]	(−0.004; −0.003)	−0.003∗∗∗ [5.9%]	(−0.003; −0.003)
Long-term health condition(s)	0.001∗∗∗ [−2.1%]	(0.001; 0.001)	0.001∗∗∗ [−1.4%]	(0.001; 0.001)
Father characteristics:				
Highest education obtained			−0.009∗ [17.5%]	(−0.009; −0.008)
Skill level of occupation			−0.002∗∗∗ [4.7%]	(−0.003; −0.002)
Hours worked the week prior to census			0.000∗∗∗ [1.0%]	(−0.001; 0.000)
Total personal income from all jobs			−0.003∗∗∗ [6.9%]	(−0.004; −0.003)
Long-term health condition(s)			0.001∗∗∗ [−1.3%]	(0.001; 0.001)
Family & household characteristics:				
Country of birth of parents	0.000∗∗∗ [−0.5%]	(0.000; 0.000)	0.000∗∗∗ [−0.2%]	(0.000; 0.000)
Family type	−0.005∗∗∗ [10.0%]	(−0.006; −0.005)		
Household size	0.000∗∗∗ [−0.5%]	(0.000; 0.000)	−0.001∗∗∗ [2.0%]	(−0.001; −0.001)
*Geographical &socio-economiccharacteristics of local living area:*				
Remoteness	0.000 [0.1%]	(0.000; 0.000)	0.000 [−0.2%]	(0.000; 0.000)
SEIFA of local living area	−0.012∗∗∗ [21.3%]	(−0.012; −0.011)	−0.009∗∗∗ [17.8%]	(−0.009; −0.008)
State of residence	−0.001∗∗∗ [1.1%]	(−0.001; 0.000)	−0.001∗∗∗ [1.2%]	(−0.001; 0.000)
Total number of children	1,237,470		955,740	
Of which:				
Public schools	963,720		719,620	
Private schools	273,750		236,120	

Note: ∗∗∗P value <0.01, ∗∗P value <0.05, and ∗P value <0.1.

Number of observations are rounded to nearest 10 per the ABS's confidentiality rules.

Estimated gaps and 95% CIs are rounded to three decimal places.

Model 1 controls for child, mother, family and residential area's characteristics.

Model 2 adds to Model 1 father's characteristics, therefore applied to children in couple parents' families only.

The explained part (overall shown in Panel B; by each observed characteristics in Panel C) is the gap due to differences in observed characteristic. The unexplained part (Panel B) is the gap due to differences in estimated coefficients and other factors not captured in the model. Please see Supplementary Material 1 for details on the Oaxaca-Blinder decomposition method.

The first two rows of Panel A show influenza vaccination coverage among children in public and private schools. The overall coverage gap between children in the two school types is shown in the first row of Panel B. The next two rows of Panel B decompose this overall gap into “explained” and “unexplained” components. The “explained” component indicates how much of the total coverage gap is attributable to differences in observed characteristics between the two groups. The “unexplained” component indicates how much of the coverage gap is due to other factors not controlled in the model. Panel C further breaks down the explained gap into the contribution of each factor.

The results from the decomposition applied to Model 1, which controls for characteristics of the child, mother, family and the local living area, show that 51% (or 0.028/0.054) of the total vaccination coverage gap between public and private secondary schools is due to differences in the observed health, linguistic, cultural, socio-economic, and demographic characteristics of children, their mothers, families, and geographical and socio-economic characteristics of local living areas. Among the contributing factors, differences in mother's education, mother's income, family type, and socio-economic characteristics of residential areas play the most important role, making 22%, 6%, 10%, and 21% contribution to the overall gap, respectively. Notably, the higher proportion of children from single-parent families in public schools contributes approximately 10% (0.005/0.054) to the overall vaccination coverage gap between children in the two school types.

The results from the decomposition applied to Model 2, which additionally controls for father's characteristics, reveals that nearly 64% (or 0.032/0.050) of total uptake gap is due to differences in the observed health, linguistic, cultural, socio-economic, and demographic characteristics of children, their parents (both mother and father), families; and geographical and socio-economic characteristics of residential areas. Among the contributing factors, consistent with the decomposition results applied to Model 1, differences in parental education, skill level of occupation, income, and socio-economics characteristics of residential area play the most important role. For example, the better education and higher income of mothers of children in private than public schools contributes 24% to the uptake gap. Of this, 18% (or 0.009/0.05) is due to mothers' better education, and 6% (or 0.003/0.05) is due to mothers' higher income. Similarly, the better education, higher skill level in occupation, and higher income of fathers of children in private schools contributes 30% to the uptake gap. Of this, 18% (or 0.009/0.05) is due to fathers' higher education, 5% (or 0.0024/0.05) is due to fathers' better skill level of occupation, and 7% (or 0.0034/0.05) is due to fathers' higher income.

In both models, difference in socio-economic characteristics of residential areas make a remarkable contribution to the uptake gap. The fact that children in private schools tend to reside in more advantaged socio-economic areas than those in public schools contributes 21% (or 0.0115/0.054) to the gap in Model 1, and 18% (or 0.009/0.05) in Model 2 (Table 2).

### Robustness check

In 2021, influenza vaccination uptake across all age groups was lower than that in 2023 except among the elderly. This was likely due to the absence of influenza activity in the community throughout 2020 and 2021, resulting from the COVID−19 public health non-pharmaceutical interventions targeted at controlling the SARS-CoV−2 transmission.^31^

In 2021, uptake among secondary school-aged children was lowest of all age groups (Supplementary Figure S1). A sizable uptake gap existed between public and private schools. While 17.2% of private secondary school students were vaccinated against influenza, coverage was just 13.9% for public schools. Higher coverage in private than public schools consistently occurred in all jurisdictions (Supplementary Figure S2).

Differences in observed characteristics made a smaller contribution to the overall uptake gap in 2021 (33.2% in Model 1 and 37.8% in Model 2) than in 2023 (51.1% in Model 1 and 63.3% in Model 2). This reduction is mostly driven by decreases in the contribution from mother's education, mother's income, and socio-economic characteristics of residential areas. Differences in how mothers/families adjust their vaccination behaviour in response to perceived influenza risk may help explain this pattern. In particular, the low influenza activity in 2021 may have reduced demand for vaccination, with this effect being more pronounced among advantaged socio-economic groups. Consistent with findings from our main analysis, in 2021, public-private schools' differences in the health, cultural, socio-economic, and demographic characteristics of children, their parents, families, and residential areas, remain to be key contributors to the gap. Among these factors, parental education, parental income, mothers' religion, family structure, and socio-economic characteristics of residential areas play the most important role (Supplementary Table S4).

## Discussion

In this study, from analysis of individually linked datasets encompassing the entire population, we showed that influenza vaccination uptake among Australian children in 2023 was low and unevenly distributed despite funded seasonal influenza vaccination (SIV) programs. Of all age groups, secondary school-aged children have the lowest uptake, with a significant uptake gap between public and private schools. Across all states and territories, influenza vaccination coverage is consistently higher among children in private than in public schools.

We found that cultural, linguistic, and socio-economic characteristics of parents and residential areas, along with long-term health conditions of children and their parents, are key predictors of children's influenza vaccination uptake. This is consistent with what was found in the literature on childhood vaccination or COVID−19 vaccine uptake in Australia, influenza vaccination uptake for adults in Europe, childhood vaccination in Scotland, influenza vaccine uptake for the whole population in Liverpool - United Kingdom (UK), and influenza vaccination uptake among the elderly in United States (US).^17^, ^18^, ^19^, ^20^, ^21^, ^22^ However, different from the literature when exploring children's influenza vaccination uptake by sub-groups classified by additional language speaks at home, we found that children who speak Southern European, Eastern European, Southwest and Central Asian languages as an additional language at home had notably lower uptake compared to children who speak English only. But children who speak Southern Asian, Southeast Asian and Eastern Asian language at home have higher uptake compared to children who speak English only. Consistent with the literature, we find that children whose mothers report an Islamic religion had significantly lower uptake than children whose mothers are Christian.^32^ Furthermore, children whose mothers reports Australian Aboriginal cultural beliefs had lower uptake compared to children whose mothers are Christian. The pattern is consistent across both public and private schools. Our finding suggests that future initiatives in the SIV campaigns should consider tailored strategies that speak to specific cultural, linguistic, and socio-economic groups.

Our decomposition results reveal that roughly two-thirds of the vaccination coverage gap between children in public and private secondary schools is due to differences in the observed health, cultural, linguistic, and socio-economic characteristics of children, their parents, families, along with geographical and socio-economic characteristics of residential areas. Among these, parental education, parental employment, and socio-economic characteristics of local living areas play the most important roles. The findings suggest that a long-term strategy with multi-sectoral approach addressing systematic socio-economic inequalities (such as parental education, and other factors relating to socio-economic status) could have a lasting impact on reducing vaccination uptake inequity for children. Short-term measures aimed at reducing disadvantage (such as improving parental employment and skill level of occupation for parents of underprevileged children and those in areas of less socio-economic advantage) could improve children's vaccination uptake and lower the uptake inequity.

The notable socio-economic disparity between public and private schools found in this study using the Census is consistent with findings from previous studies using the nationally representative Longitudinal Study of Australian Children,^33^ and mirrors results from a global educational report which identifies Australia as having the second-highest social segregation between public and private schools among OECD countries.^23^ This should not be interpreted as an evidence of a broken system or failed public policies. Disparity exists in every society where people differ in talents, preferences, and aspirations. However, disparity in Australia is further heightened by a long history of immigration. In fact, per student Australian government spending for public schools is 60% higher than that for private schools, but Australian parents of children in private schools make significant financial contributions toward school income.^34^ This results in a marked difference in the amount of money that private schools can spend per student than public schools. Students in private schools are not only more likely to have access to better facilities, better study resources, and higher quality teachers to realise their full potential, but, as our study shows, they also tend to come from families with more advantaged socio-economic backgrounds. Given that public schools serve a larger proportion of children from less advantaged socio-economic backgrounds, discrepancy in vaccination coverage may lead to unequal opportunities for protection against serious consequences of influenza infection, maintaining school attendance, and participating in other developmental activities. This may further deepen any existing health and social-economic divides. Similar to what was found in the literature on childhood vaccination uptake in Scotland,^18^ our finding for Australia indicates that resources that have been targeted at improving Australian children's health may not have sufficiently reached and benefited the most disadvantaged groups. Our finding suggests that public schools should be a key focus in Australia's future SIV campaigns.

Even with the higher influenza vaccination coverage among Australian private school children (of 19.6% in 2023), coverage remains significantly lower than that for children in the UK (42%) and the US (50%).^35^^,^^36^ A possible explanation is that while the UK has a school-based fully funded SIV program, such a school-based SIV program has not been available in Australia. Furthermore, while only the Inactivated Influenza Vaccines (IIV) administered via injection are available in Australia, both IIV and the live attenuated influenza vaccine (LAIV) administered via nasal spay are available in the UK and the US.^37^ With influenza cases reaching a record high,^4^ boosting influenza vaccination coverage in the population and among school-aged children is needed. Alternative methods of SIV delivery for school-aged Australian children, such as LAIV in a nasal spray and a school-based program, to improve influenza vaccination uptake could be explored.

To our knowledge, this is the first study to explore inequity in influenza vaccination uptake between public and private schools, and apply a robust statistical decomposition method to pinpoint the absolute and relative importance of factors behind the uptake inequity. By analysing whole-of-population data and leveraging novel features of the AIR system, which accurately records all vaccines administered to the population, we overcome possible limitations in previous studies. These include biases due to under-reporting of influenza vaccines, sample selection, and population non-representativeness in survey data. Further more, by utilising the unique features of the Australian Census, we were able to link children with their parents. Therefore, for the first time in the literature, we capture a rich set of health, cultural, linguistic, socio-economic, and demographic characteristics of both children and their parents.

While making novel contributions to the literature, our study could be improved by addressing the following limitations. First, we were able to link 95% of children with their mothers and 80% with their fathers using relationships identified in the Census, but not all children. This is because family identification in Census is nested within dwelling so if a mother or father lived elsewhere, we were unable to capture it. Future research should utilise the Core Relationship among Australians data, produced by the ABS, when it becomes available to achieve higher linkage rates for mothers, fathers, and children than in our current work. This Core Relationship data will have relationship captured not only from Censuses but also other administrative sources. Second, precision of our study could also be improved by strengthening the linkage between Medicare and Census. Third, here we quantify the extent to which socio-economic inequality translates into SIV uptake inequity at a point in time. Due to confidentiality rules, linking entire data from multiple Censuses is currently not possible. Future research could apply for the Census Longitudinal Dataset, a 5% sample of the Census, to be integrated with the AIR. This would allow further exploration of how changes in socio-economic inequality and shifts in population structure over time change SIV uptake inequity. Additionally, evaluating effectiveness of state-funded SIV policies for school-aged children on improving SIV uptake and reducing this inequity should also be explored.

In conclusion, using the whole-of-population data, we investigated influenza vaccination uptake among school-aged Australian children in 2023, explored the vaccination uptake gap between public and private schools, and provided new insights into the extent to which structural socio-economic inequality shape vaccination uptake inequity. Our findings highlight the need for tailored strategies in vaccination campaigns, and a multi-sectoral approach addressing the existing structural socio-economic inequality to reduce vaccination uptake inequity in children attending public and private schools.

## Contributors

HL, CCB, and HCM conceived the study. HL conducted the analyses and drafted the manuscript with input from CCB, HCM, HN, and BL. HL, HCM, JM, FM, RF, SM, CH, and CCB acquired funding. This study is part of the project “Understanding Australia's Immunisation Programs” of Australian Department of Health and Aged Care (DOHAC) led by CS. CS, CCB, HCM, BL, RF, and HL had full access to all the data. All authors contributed to refine the interpretation and review draft versions the manuscript. All authors accepted responsibility to submit the manuscript for publication.

## Data sharing statement

This project uses de-identified administrative person record level data. The data is part of broader data set in the Australian Government–DOHAC's “Understanding Australia's Immunisation Programs” project. The data is administered by the ABS in partnership with DOHAC and other related Australian Government Agencies. The study authors accessed and analysed the de-identified data remotely via a virtual machine in administered by the ABS. Access to data is restricted to named researchers only. Analytical codes are available from the corresponding author upon request. However, the study authors do not own data. Request for access to the data should be directed to the Australian Government's DOHAC and the ABS.

## Declaration of interests

The authors declare the following financial interests/personal relationships which may be considered as potential competing interests: HL, CCB, BL, FM, and HN report grants support provided by National Health and Medical Research Council. FM and HN report financial support provided by Australian Research Council. CH is executive director of the Immunisation Foundation of Australia, a non-profit organisation which has received educational grants for immunisation-related projects and campaigns (Pfizer, Sanofi, MSD, GSK), but these were not specifically related to influenza or this study. HCM has received travel support to attend a real-world influenza vaccine policy summit sponsored by Seqirus and was a member of the Independent Scientific Steering Committee; has received institutional honoraria for participation in advisory boards from Merk Sharpe and Dohme (Australia), GSK, Sanofi, Pfizer and EvoHealth; none of these are related to the work reported in this manuscript.

## Footnotes

n The term inequity refers to disparity or difference in an unfair way.
