# Supplementary material for: Socio-economic inequality underpins inequity in influenza vaccination uptake between public and private secondary schools: an Australian population-based study
Source: Lancet Reg Health West Pac. 2025 Nov 28;65:101761. doi: 10.1016/j.lanwpc.2025.101761 (PMC12702385; doi:10.1016/j.lanwpc.2025.101761)
Supplement: Supplementary Materials [file mmc1.docx]

**Supplementary Information**

**Supplementary Material 1: Further details on the Oaxaca-Blinder decomposition, logistic regression model specification, and identification of secondary school-aged children**

The Oaxaca-Blinder decomposition^1, 2^ involves the following steps:

Step 1: Comparing characteristics of secondary school-aged children, their parents (separately for mothers and fathers), families, and residential areas across school types.

Step 2: Estimating logistic regression models to identify important predictors for influenza vaccination uptake in children for each school-type.

Our dependent variable in the logistic regression model in Step 2 is children’s influenza vaccination status, which is in a binary form (=1 if received influenza vaccine; =0 if not).

Our set of independent variables in the model includes the health, linguistic, religion, cultural, socio-economic, and demographic characteristics of children, their parents, families and local living areas. These variables were derived from the Census.

For example, a binary variable indicating the presence of at least one long-term health condition was generated separately for children, their mother, and father based on their responses to relevant Census questions. As another example, following the ABS’s *Standard Classification of Religious Groups*, religious affiliations reported in the Census (151 categories) were classified in to eight major groups: Christianity, Buddhism, Hinduism, Islam, Judaism, Australian Aboriginal cultural belief and Other religions, and No religion or spiritual belief.^3^ Similarly, following the ABS’s *Standard Classification of Languages*, additional languages used at home (449 categories) were classified into nine major groups: Northern European (including English), Southern European, Eastern European, Southwest and Central Asian, Southern Asian, Southeast Asian, Eastern Asian, Australian Indigenous Languages, and Other languages.^4^ Furthermore, the 321 ancestry categories (first response) were also grouped into one of nine major groups based on the *ABS’s Standard Classification of Cultural and Ethnic Groups*.^5^ A list of characteristics relating to children, their mothers and fathers is provided in Supplementary Table 4.

Because we observed a rich set of comparable variables for children, their mothers, and fathers; and that these variables are highly correlated, we checked for a potential multicollinearity problem via the variance inflation factor (VIF). Variables with VIF>5 were excluded from our model. All variables with VIF≤5 were retained. Likelihood ratio tests were used to determine the set of variables included in the final model. For each observed characteristic with *n* categories, *(n-1)* binary variables were generated.

Model 1 controls for child, mother and family characteristics, and applies to entire children in the population cohort. Model 2 builds on Model 1 adding father’s characteristics and, therefore, applies only to children in couple-parent families.

Step 3: Using results from Steps 1 and 2, the difference in influenza vaccine uptake between children in public and private secondary schools is decomposed as follows:

$\bar{Y}_{A}-\bar{Y}_{B}=\left( \alpha_{A}+\beta_{A}\bar{X}_{A} \right)- \left( \alpha_{B}+\beta_{B}\bar{X}_{B} \right)$ (eq. 1)

$\underset{Total gap}{\underbrace{\bar{Y}_{A}-\bar{Y}_{B}}} =\underset{Explained part}{\underbrace{\beta_{A}\left( \bar{X}_{A}-\bar{X}_{B} \right)}}+ \underset{Unexplained part}{\underbrace{\left( \alpha_{A}-\alpha_{B} \right)+\bar{X}_{B}\left( \beta_{A}-\beta_{B} \right)}}$ (eq. 2)

Where:

A and B denote for the two groups of children in public and private schools, respectively.

$Y_{A}$ is SIV coverage in public school children (group A), and $Y_{B}$ is influenza vaccination coverage in private school children (group B).

$X_{A}$ and $X_{B}$ are vectors of explanatory variables observed at child, mother, father, family, and local living area for public and private school children, respectively.

$\beta_{A}$ and $\beta_{B}$ are vectors of the estimated coefficients from the logistic regression models, and $\alpha_{A}$ and $\alpha_{B}$ are constant terms from the regression models for public and private school children.

Because the decomposition (in eq. 2) is sensitive to the choice of the reference group,^6, 7^ to overcome the problem, we use vector of estimated coefficients $\beta^{*}$ from a pooled sample of both groups as the reference.^8^ The decomposition is now written as:

$\underset{Total gap}{\underbrace{\bar{Y}_{A}-\bar{Y}_{B}}} =\underset{Explained part}{\underbrace{\beta^{*}\left( \bar{X}_{A}-\bar{X}_{B} \right)}}+\underset{Unexplained part}{\underbrace{{\left( \alpha_{A}-\alpha_{B} \right)+\bar{X}}_{A}\left( \beta_{A}-\beta^{*} \right)+\bar{X}_{B}\left( \beta^{*}-\beta_{B} \right)}}$ (eq. 3)

Total gap of influenza vaccination coverage between children in public and private schools is decomposed into two parts: (i) a part which is due to differences in observed characteristics, called an explained part, and (ii) another part which is due to differences in the estimated coefficient and other factors not controlled for in the model, called an unexplained part.

In this study, our outcome of interest is binary, which departs from the traditional continuous form. Therefore, the logistic regression model was used in Step 2. The Oaxaca-Blinder decomposition applied to logistic regression model was used to quantify relative importance of factors contributing to differences in influenza vaccine uptake between children in public and private schools.^9-11^ Below we describe in detail the Oaxaca-Blinder decomposition with application to logistic models.

Suppose that a dependent variable is a function of a linear combination of independent variables through a mapping function $F\left( . \right)$. The function $F\left( . \right)$ itself may or may not be linear but is differentiable: $Y=F\left( X\beta\right)$.

The difference in the outcome Y of the two groups (A and B) at the first moment (the mean difference of Y between groups A and B) is decomposed as:

$\underset{Total gap}{\underbrace{\bar{Y}_{A}-\bar{Y}_{B}}}=\underset{Explained part}{\underbrace{\left[ \bar{F\left( X_{A}\beta_{A} \right)}-\bar{F\left( X_{B}\beta_{A} \right)} \right]}}+\underset{Unexplained part}{\underbrace{\left[ \bar{F\left( X_{B}\beta_{A} \right)}-\bar{F\left( X_{B}\beta_{B} \right)} \right]}}$ (eq. 4)

When observed outcome of the two groups are in continuous form, general linear regression models are used in Step 2. Because in GLM (i.e., ordinary least square regression - OLS): $\bar{F\left( X \right)}=F\left( \bar{X} \right)$ (*), as such in traditional Oaxaca-Blinder decomposition, the two components in (eq. 4) can easily be obtained.

However, when outcome departs from the continuous form, the estimation is done through a latent variable (i.e., transformed via the logit link function), and this transformation function is not linear so (*) does not hold. We can not directly obtain the two explained & unexplained components in (eq. 4).

Yun (2004) proposed a detailed decomposition that extends the traditional Oaxaca-Blinder decomposition approach to accommodate flexible regression models.^9^ The method involves constructing proper weights to the characteristics and coefficients effects through first order Taylor expansion to linearize the characteristics and coefficients effects:

$$\bar{Y}_{A}-\bar{Y}_{B}=\left[ F\left( \bar{X}_{A}\beta_{A} \right)-F\left( \bar{X}_{B}\beta_{B} \right) \right]+\left[ F\left( \bar{X}_{B}\beta_{A} \right)-F\left( \bar{X}_{B}\beta_{B} \right) \right] +R_{M}$$

$\bar{Y}_{A}-\bar{Y}_{B}=\left[ \left( \bar{X}_{A}-\bar{X}_{B} \right)\beta_{A} \right]f\left( {\bar{X}_{A}\beta}_{A} \right)+\bar{X}_{B}\left( \beta_{A}-\beta_{B} \right)f\left( {\bar{X}_{B}\beta}_{B} \right)+ R_{M}+R_{T}$ (eq. 5)

Where:

$R_{M}$ and $R_{T}$are approximation residuals resulting from evaluating the function $F\left( . \right)$ at the mean values and using the first order Talyor expansion, respectively.

$$R_{M}=\left[ \bar{F\left( X_{A}\beta_{A} \right)}-\bar{F\left( X_{B}\beta_{A} \right)} \right]+\left[ \bar{F\left( X_{B}\beta_{A} \right)}-\bar{F\left( X_{B}\beta_{B} \right)} \right]-\left[ F\left( \bar{X}_{A}\beta_{A} \right)-F\left( \bar{X}_{B}\beta_{A} \right) \right]-\left[ F\left( \bar{X}_{B}\beta_{A} \right)-F\left( \bar{X}_{B}\beta_{B} \right) \right]$$

$$R_{T}=\left[ F\left( \bar{X}_{A}\beta_{A} \right)-F\left( \bar{X}_{B}\beta_{A} \right) \right]+\left[ F\left( \bar{X}_{B}\beta_{A} \right)-F\left( \bar{X}_{B}\beta_{B} \right) \right]-\left[ \left( \bar{X}_{A}-\bar{X}_{B} \right)\beta_{A} \right]f\left( \bar{X}_{A}\beta_{A} \right)-\bar{X}_{B}\left( \beta_{A}-\beta_{B} \right)f\left( \bar{X}_{B}\beta_{B} \right)$$

$f\left( \bar{X}_{j}\beta_{j} \right)=\frac{dF\left( \bar{X}_{j}\beta_{j} \right)}{d\left( \bar{X}_{j}\beta_{j} \right)}$ that is $f\left( . \right)$is the first-order derivative of the function $F\left( . \right)$, j is group indicator (=A or B)

A detailed decomposition of (eq. 5) is written as:

$\bar{Y}_{A}-\bar{Y}_{B}=\sum_{i=1}^{i=K} W_{\Delta X}^{i}\left[ \bar{F\left( X_{A}\beta_{A} \right)}-\bar{F\left( X_{B}\beta_{A} \right)} \right]+\sum_{i=1}^{i=K} W_{\Delta\beta}^{i}\left[ \bar{F\left( X_{B}\beta_{A} \right)}-\bar{F\left( X_{B}\beta_{B} \right)} \right]$ (eq. 6)

Where:

$W_{\Delta X}^{i}$ and $W_{\Delta\beta}^{i}$ are the weights attached to a variable (i), which can be calculated using the mean values of characteristics and coefficients as follow:

$$W_{\Delta X}^{i}=\frac{\left( \bar{X}_{A}^{i}-\bar{X}_{B}^{i} \right)\beta_{A}^{i}f\left( \bar{X}_{A}\beta_{A} \right)}{\left( \bar{X}_{A}-\bar{X}_{B} \right)\beta_{A}f\left( \bar{X}_{A}\beta_{A} \right)}=\frac{\left( \bar{X}_{A}^{i}-\bar{X}_{B}^{i} \right)\beta_{A}^{i}}{\left( \bar{X}_{A}-\bar{X}_{B} \right)\beta_{A}}$$

$$W_{\Delta\beta}^{i}=\frac{\bar{X}_{B}^{i}\left( \beta_{A}^{i}-\beta_{B}^{i} \right)f\left( \bar{X}_{B}\beta_{B} \right)}{\bar{X}_{B}\left( \beta_{A}-\beta_{B} \right)f\left( \bar{X}_{B}\beta_{B} \right)}=\frac{\bar{X}_{B}^{i}\left( \beta_{A}^{i}-\beta_{B}^{i} \right)}{\bar{X}_{B}\left( \beta_{A}-\beta_{B} \right)}$$

in which:

$$\sum_{i=1}^{i=K} W_{\Delta X}^{i}=\sum_{i=1}^{i=K} W_{\Delta\beta}^{i}=1$$

It should be noted that the Oaxaca-Blinder decomposition is valid under the assumption of correct model specification in the logistic regression. Furthermore, the results may be sensitive to the choice of reference group when categorical variables are included.^12^ Therefore, the results should be interpreted with caution.

The Oaxaca-Blinder decomposition method was first developed in the field of labour economics, for studies on labour market discrimination, where the outcome of interest is continuous. Therefore, the ordinary least square estimation method was used in Step 2. For example, Oaxaca^1^ (1973) studied male-female wage differential in urban labour markets, and Blinder^2^ (1973) investigated black-white wage differential among male workers to quantify the extent to which workers with similar characteristics but received different pay. In labour economics, the “unexplained part” is called “discrimination”. The Oaxaca-Blinder decomposition method is now widely applied in other fields.^13-17^

Since secondary school-aged children have the lowest influenza vaccination coverage and the largest coverage gap between children attending public and private schools, our logistic regression and Oaxaca-Blinder decomposition focus on children in this group.

To capture the most accurate population cohort of secondary school-aged children in 2023, we derived children’s ages based on the cut-off for school-starting age (turning 5 years old) as set by the jurisdiction in which they lived: 1 Jan for Tasmania (TAS); 30 Apr in the Australian Capital Territory (ACT), Victoria (VIC) and South Australia (SA); 30 Jun in Queensland (QLD), Western Australia (WA) and Northern Territory (NT); and 31 Jul in New South Wales (NSW). Secondary school-aged children include those aged 12-18 years in the ACT, NSW, VIC, WA, TAS, and NT; and those aged 13-18 years in QLD and SA. We set a different starting age for secondary school-aged children due to variations in secondary school entry years across Australian states and territories. Specifically, secondary school education begins in Year 7 (12 years old) in ACT, NSV, VIC, WA, TAS, and NT; but it starts in Year 8 (13 years old) in QLD and SA.

**Supplementary Table 1: Influenza vaccination coverage (%) by age-groups and jurisdictions in 2023**

| **Age groups** | **ACT** | **NSW** | **VIC** | **QLD** | **SA** | **WA** | **TAS** | **NT** | **All Australia** |
| --- | --- | --- | --- | --- | --- | --- | --- | --- | --- |
|  |  |  |  |  |  |  |  |  |  |
| **6mths-<5yrs** | 49.65 | 26.66 | 31.92 | 24.06 | 28.16 | 27.19 | 33.08 | 38.07 | **28.21** |
| **5-12yrs** | 27.47 | 16.62 | 18.83 | 15.81 | 16.94 | 19.89 | 17.33 | 16.83 | **17.59** |
| **12-<18yrs** | 19.39 | 13.29 | 15.26 | 14.02 | 14.64 | 16.68 | 14.86 | 17.89 | **14.57** |
| **18-<50yrs** | 34.76 | 22.18 | 26.55 | 21.93 | 26.92 | 22.96 | 26.30 | 25.64 | **23.98** |
| **50-<65yrs** | 47.04 | 35.02 | 39.37 | 37.00 | 41.53 | 38.02 | 44.99 | 29.48 | **37.64** |
| **>=65yrs** | 69.30 | 62.42 | 66.07 | 65.71 | 70.21 | 66.15 | 72.22 | 39.05 | **65.13** |
|  |  |  |  |  |  |  |  |  |  |
| **Overall** | **41.28** | **31.23** | **34.67** | **31.30** | **37.05** | **32.46** | **38.80** | **27.35** | **32.91** |

Note: ACT is abbreviated for Australian Capital Territory; NSW is for New South Wales; VIC is for Victoria; QLD is for Queensland; SA is for South Australia; WA is for Western Australia; TAS is for Tasmania; and NT is for Northern Territory.

**Supplementary Table 2: Influenza vaccination coverage (%) by school types, education levels, and jurisdictions in 2023**

| Education levels ^ϯ^ | School types**^ͱ^** | | | | Overall |
| --- | --- | --- | --- | --- | --- |
|  | **Public** | **Private** | **Catholic** | **Not stated** |  |
| Secondary school |  |  |  |  |  |
| ACT | 19.28 | 24.89 | 21.05 | 16.67 | 20.55 |
| NSW | 13.39 | 17.65 | 15.37 | 9.66 | 14.42 |
| VIC | 14.84 | 21.56 | 17.41 | 11.87 | 16.48 |
| QLD | 12.90 | 19.52 | 19.96 | 11.47 | 15.40 |
| SA | 13.93 | 18.94 | 16.92 | 11.59 | 15.45 |
| WA | 16.78 | 20.60 | 21.25 | 12.29 | 18.19 |
| TAS | 14.26 | 20.04 | 18.07 | 10.83 | 15.78 |
| NT | 14.25 | 15.77 | 14.78 | 25.00 | 15.14 |
| Overall sec. schools | **14.22** | **19.62** | **17.61** | **11.38** | **15.78** |
|  |  |  |  |  |  |
| Primary school ^ƪ^ |  |  |  |  |  |
| ACT | 25.83 | 30.42 | 26.20 | 17.14 | 26.29 |
| NSW | 16.38 | 17.70 | 18.39 | 12.08 | 16.81 |
| VIC | 18.28 | 20.58 | 20.48 | 13.05 | 18.84 |
| QLD | 15.47 | 18.25 | 21.00 | 11.72 | 16.71 |
| SA | 16.24 | 18.11 | 18.28 | 13.21 | 16.83 |
| WA | 20.21 | 19.32 | 24.68 | 14.97 | 20.68 |
| TAS | 16.90 | 18.18 | 18.52 | 11.43 | 17.15 |
| NT | 13.62 | 10.48 | 18.69 | 20.00 | 14.12 |
| Overall prim. schools | **17.14** | **18.83** | **20.26** | **12.67** | **17.81** |
|  |  |  |  |  |  |
| Overall prim. & sec. schools | **15.56** | **19.36** | **18.66** | **11.98** | **16.65** |

Notes: **^ͱ^** School type is from the 2021 Census

**^ϯ^** Education level is identified via children’s age.

**^ƪ^** Primary school includes children from 8 years old because we consider flu vaccines administered in 2023 but use school-type reported in the 2021 Census. Therefore, school type is unavailable for children who started pre-school or Year1 in 2022 or 2023.

**Supplementary Table 3: Comparative characteristics of secondary school-aged children, their parents, families, and residential areas for those in public vs. private schools**

|  | **Public^¶^** | **Private^¶^** | **Test for diff.^Ŧ^**  **P value** |
| --- | --- | --- | --- |
|  |  |  |  |
| **Total number of children (%)** | **963,720 (60.9%)** | **273,750 (17.3%)** |  |
|  |  |  |  |
| **Child characteristics:** |  |  |  |
| *Age* | 15.030 (1.705) | 15.353 (1.622) | <0.001 |
|  |  |  |  |
| *Gender* |  |  |  |
| Male | 500,740 (52.0%) | 136,690 (49.9%) | <0.001 |
| Female | 462,980 (48.0%) | 137,060 (50.1%) |  |
|  |  |  |  |
| *Aboriginal and/or Torres Strait Islander* |  |  |  |
| No | 902,810 (93.7%) | 266,600 (97.4%) | <0.001 |
| Yes | 55,590 (5.8%) | 5,650 (2.1%) |  |
| NA** or missing | 5,320 (0.6%) | 1,500 (0.5%) |  |
|  |  |  |  |
| *Australian citizenship* |  |  |  |
| Australian | 895,360 (92.9%) | 260,900 (95.3%) | <0.001 |
| Not Australian | 65,170 (6.8%) | 12,100 (4.4%) |  |
| NA** or missing | 3,190 (0.3%) | 750 (0.3%) |  |
|  |  |  |  |
| *Long-term health condition(s)* |  |  |  |
| No long-term health condition | 739,810 (76.8%) | 218,710 (79.9%) | <0.001 |
| >=1 long-term health condition | 183,140 (19.0%) | 45,600 (16.7%) |  |
| NA or missing | 40,770 (4.2%) | 9,440 (3.4%) |  |
|  |  |  |  |
| *Additional language speaks at home* |  |  |  |
| Northern European including English | 779,260 (80.9%) | 223,160 (81.5%) | <0.001 |
| Southern European | 12,490 (1.3%) | 4,630 (1.7%) |  |
| Eastern European | 9,700 (1.0%) | 2,230 (0.8%) |  |
| Southwest & Central Asian | 34,110 (3.5%) | 11,130 (4.1%) |  |
| Southern Asian | 43,050 (4.5%) | 12,030 (4.4%) |  |
| Southeast Asian | 27,010 (2.8%) | 3,510 (1.3%) |  |
| Eastern Asian | 34,280 (3.6%) | 11,970 (4.4%) |  |
| Australian Indigenous Languages | 3,630 (0.4%) | 460 (0.2%) |  |
| Other languages | 11,740 (1.2%) | 3,020 (1.1%) |  |
| NA or missing | 8,460 (0.9%) | 1,620 (0.6%) |  |
|  |  |  |  |
| *Religion* |  |  |  |
| Christianity | 317,690 (33.0%) | 135,220 (49.4%) | <0.001 |
| Buddhism | 20,360 (2.1%) | 2,580 (0.9%) |  |
| Hinduism | 26,600 (2.8%) | 5,280 (1.9%) |  |
| Islam | 51,790 (5.4%) | 20,940 (7.6%) |  |
| Judaism | 2,310 (0.2%) | 4,830 (1.8%) |  |
| Aus. Aboriginal cultural beliefs or other religions | 13,820 (1.4%) | 2,850 (1.0%) |  |
| No religion or secular belief | 514,020 (53.3%) | 97,430 (35.6%) |  |
| NA or missing | 17,140 (1.8%) | 4,630 (1.7%) |  |
|  |  |  |  |
| *Country of birth of parents* |  |  |  |
| Both parents born in Australia | 545,740 (56.6%) | 142,430 (52.0%) | <0.001 |
| One parent born oversea | 159,430 (16.5%) | 55,430 (20.2%) |  |
| Both parents born oversea | 252,940 (26.2%) | 75,120 (27.4%) |  |
| NA or missing | 5,620 (0.6%) | 770 (0.3%) |  |
|  |  |  |  |
| **Mother’s characteristics:** |  |  |  |
| *Age of mother ^‽^* | 45.218 (6.441) | 47.132 (5.573) | <0.001 |
|  |  |  |  |
| *Ancestry by major group-1st response* |  |  |  |
| Oceania including Aus, NZ & Islands surrounded | 361,180 (37.5%) | 90,160 (32.9%) | <0.001 |
| North-West Europe | 379,990 (39.4%) | 114,850 (42.0%) |  |
| Southern & Eastern Europe | 43,430 (4.5%) | 13,860 (5.1%) |  |
| North Africa & Middle East | 27,780 (2.9%) | 10,910 (4.0%) |  |
| South-East Asia | 32,520 (3.4%) | 4,670 (1.7%) |  |
| North-East Asia | 46,240 (4.8%) | 16,920 (6.2%) |  |
| Southern & Central Asia | 59,330 (6.2%) | 17,390 (6.4%) |  |
| Americas | 3,820 (0.4%) | 1,120 (0.4%) |  |
| Others | 4,410 (0.5%) | 1,820 (0.7%) |  |
| NA or missing | 5,040 (0.5%) | 2,060 (0.8%) |  |
|  |  |  |  |
| *Australian citizenship status* |  |  |  |
| Australian | 788,940 (81.9%) | 234,620 (85.7%) | <0.001 |
| Not Australian | 111,250 (11.5%) | 23,420 (8.6%) |  |
| NA or missing | 63,540 (6.6%) | 15,710 (5.7%) |  |
|  |  |  |  |
| *Registered marital status* |  |  |  |
| Married | 587,690 (61.0%) | 213,420 (78.0%) | <0.001 |
| Separated, Divorce or Widowed | 155,360 (16.1%) | 29,370 (10.7%) |  |
| Never married | 159,130 (16.5%) | 15,720 (5.7%) |  |
| NA or missing | 61,540 (6.4%) | 15,240 (5.6%) |  |
|  |  |  |  |
| *Proficiency in spoken English* |  |  |  |
| Speaks English only | 724,290 (75.2%) | 204,020 (74.5%) | <0.001 |
| English very well & use other language | 199,150 (20.7%) | 63,480 (23.2%) |  |
| English not well & other language | 40,280 (4.2%) | 6,250 (2.3%) |  |
|  |  |  |  |
| *Religious affiliation* |  |  |  |
| Christianity | 369,570 (38.3%) | 138,820 (50.7%) | <0.001 |
| Buddhism | 31,140 (3.2%) | 5,240 (1.9%) |  |
| Hinduism | 27,000 (2.8%) | 5,520 (2.0%) |  |
| Islam | 50,880 (5.3%) | 20,660 (7.5%) |  |
| Judaism | 2,440 (0.3%) | 4,650 (1.7%) |  |
| Aus. Aboriginal cultural beliefs or other religions | 15,570 (1.6%) | 2,880 (1.1%) |  |
| No religion or secular belief | 392,250 (40.7%) | 77,300 (28.2%) |  |
| NA or missing | 74,870 (7.8%) | 18,700 (6.8%) |  |
|  |  |  |  |
| *Highest education obtained* |  |  |  |
| Post graduate level | 62,420 (6.5%) | 35,250 (12.9%) | <0.001 |
| Graduate diploma & grad. certificate | 35,310 (3.7%) | 16,150 (5.9%) |  |
| Bachelor degree | 193,140 (20.0%) | 93,590 (34.2%) |  |
| Advanced diploma & diploma | 145,940 (15.1%) | 39,840 (14.6%) |  |
| Certificate I to IV levels | 167,130 (17.3%) | 25,900 (9.5%) |  |
| Years10-12 | 232,150 (24.1%) | 38,640 (14.1%) |  |
| Years 9 & below | 33,440 (3.5%) | 3,000 (1.1%) |  |
| NA or missing | 94,210 (9.8%) | 21,370 (7.8%) |  |
|  |  |  |  |
| *Occupational group* |  |  |  |
| Managers & Professionals | 244,230 (25.3%) | 115,540 (42.2%) | <0.001 |
| Technicians, Machinery Operators, Trade Workers, Admin & Clerical & other Laborers | 399,100 (41.4%) | 83,970 (30.7%) |  |
| NA or missing | 320,390 (33.2%) | 74,240 (27.1%) |  |
|  |  |  |  |
| *Skill level of occupation* |  |  |  |
| Level 1 (the highest skill) | 216,910 (22.5%) | 106,680 (39.0%) | <0.001 |
| Level 2 | 89,380 (9.3%) | 28,440 (10.4%) |  |
| Level 3 | 66,750 (6.9%) | 15,430 (5.6%) |  |
| Leve l4 | 180,230 (18.7%) | 38,220 (14.0%) |  |
| Level 5 (the lowest skill) | 90,060 (9.3%) | 10,740 (3.9%) |  |
| NA or missing | 320,390 (33.2%) | 74,240 (27.1%) |  |
|  |  |  |  |
| *Labor force part. & emp. status* |  |  |  |
| Employed, worked full-time | 335,460 (34.8%) | 109,430 (40.0%) | <0.001 |
| Employed, worked part-time | 316,090 (32.8%) | 92,590 (33.8%) |  |
| Unemployed | 30,820 (3.2%) | 5,590 (2.0%) |  |
| Not in the labour force | 216,300 (22.4%) | 50,230 (18.3%) |  |
| NA or missing | 65,060 (6.8%) | 15,920 (5.8%) |  |
|  |  |  |  |
| *Working sector* |  |  |  |
| National Government | 26,490 (2.7%) | 8,300 (3.0%) | <0.001 |
| State/Territory/Local Govt. | 128,740 (13.4%) | 34,470 (12.6%) |  |
| Private sector | 490,960 (50.9%) | 158,000 (57.7%) |  |
| NA or missing | 317,540 (32.9%) | 72,980 (26.7%) |  |
|  |  |  |  |
| *Total personal income* |  |  |  |
| <= $64.999/year | 635,520 (65.9%) | 135,620 (49.5%) | <0.001 |
| $65.000-$103.999/year | 166,690 (17.3%) | 56,960 (20.8%) |  |
| >=104.000/year | 90,690 (9.4%) | 63,670 (23.3%) |  |
| NA or missing | 70,840 (7.4%) | 17,510 (6.4%) |  |
|  |  |  |  |
| *Hours worked in the week before Census* | 19.935 (18.339) | 23.087 (18.992) | <0·001 |
|  |  |  |  |
| *Long-term health condition(s)* |  |  |  |
| No long-term health condition | 570,190 (59.2%) | 178,130 (65.1%) | <0.001 |
| >=1 long-term health condition | 304,280 (31.6%) | 72,890 (26.6%) |  |
| NA or missing | 89,260 (9.3%) | 22,740 (8.3%) |  |
|  |  |  |  |
| **Father’s characteristics:** |  |  |  |
| *Age of father^‽^* | 48.106 (7.140) | 49.719 (6.234) | <0.001 |
|  |  |  |  |
| *Australian citizenship status* |  |  |  |
| Australian | 623,770 (64.7%) | 205,140 (74.9%) | <0.001 |
| Not Australian | 87,960 (9.1%) | 19,990 (7.3%) |  |
| NA or missing | 252,000 (26.1%) | 48,620 (17.8%) |  |
|  |  |  |  |
| *Registered marital status* |  |  |  |
| Married | 569,030 (59.0%) | 205,860 (75.2%) | <0.001 |
| Separated, Divorce or Widowed | 54,330 (5.6%) | 9,710 (3.5%) |  |
| Never married | 90,010 (9.3%) | 10,020 (3.7%) |  |
| NA or missing | 250,350 (26.0%) | 48,160 (17.6%) |  |
|  |  |  |  |
| *Proficiency in spoken English* |  |  |  |
| Speaks English only | 771,050 (80.0%) | 215,540 (78.7%) | <0.001 |
| English very well & use other language | 167,880 (17.4%) | 54,290 (19.8%) |  |
| English not well/not at all & other languages | 24,580 (2.6%) | 3,890 (1.4%) |  |
|  |  |  |  |
| *Religious affiliation* |  |  |  |
| Christianity | 268,290 (27.8%) | 112,820 (41.2%) | <0.001 |
| Buddhism | 19,550 (2.0%) | 3,500 (1.3%) |  |
| Hinduism | 25,630 (2.7%) | 5,240 (1.9%) |  |
| Islam | 42,660 (4.4%) | 18,290 (6.7%) |  |
| Judaism | 2,110 (0.2%) | 4,380 (1.6%) |  |
| Aus. Aboriginal cultural belief or other religions | 14,770 (1.5%) | 3,110 (1.1%) |  |
| No religion or secular belief | 329,670 (34.2%) | 75,310 (27.5%) |  |
| NA or missing | 261,040 (27.1%) | 51,100 (18.7%) |  |
|  |  |  |  |
| *Highest education obtained* |  |  |  |
| Post graduate level | 61,390 (6.4%) | 37,710 (13.8%) | <0.001 |
| Graduate Diploma & grad. certificate | 16,260 (1.7%) | 9,200 (3.4%) |  |
| Bachelor degree | 125,360 (13.0%) | 69,080 (25.2%) |  |
| Advanced diploma & diploma | 86,420 (9.0%) | 29,290 (10.7%) |  |
| Certificate I to IV levels | 207,140 (21.5%) | 41,690 (15.2%) |  |
| Years10-12 | 162,620 (16.9%) | 30,710 (11.2%) |  |
| Years 9 & below | 30,710 (3.2%) | 3,130 (1.1%) |  |
| NA or missing | 273,830 (28.4%) | 52,950 (19.3%) |  |
|  |  |  |  |
| *Occupation group* |  |  |  |
| Managers & Professionals | 244,720 (25.4%) | 131,020 (47.9%) | <0.001 |
| Technicians, Machinery Operators, Trade Workers, Admin & Clerical & other Laborers | 359,500 (37.3%) | 72,140 (26.4%) |  |
| NA or missing | 359,500 (37.3%) | 70,590 (25.8%) |  |
|  |  |  |  |
| *Skill level of occupation* |  |  |  |
| Level 1 (the highest skill) | 216,840 (22.5%) | 119,570 (43.7%) | <0.001 |
| Level 2 | 72,930 (7.6%) | 25,170 (9.2%) |  |
| Level 3 | 120,350 (12.5%) | 25,290 (9.2%) |  |
| Leve l4 | 132,820 (13.8%) | 23,330 (8.5%) |  |
| Level 5 (the lowest skill) | 61,280 (6.4%) | 9,810 (3.6%) |  |
| NA or missing | 359,500 (37.3%) | 70,590 (25.8%) |  |
|  |  |  |  |
| *Labor force part. & emp. status* |  |  |  |
| Employed, worked full-time | 526,460 (54.6%) | 180,390 (65.9%) | <0.001 |
| Employed, worked part-time | 88,560 (9.2%) | 25,890 (9.5%) |  |
| Unemployed, looking for work | 18,970 (2.0%) | 3,810 (1.4%) |  |
| Not in the labour force | 76,660 (8.0%) | 14,960 (5.5%) |  |
| NA or missing | 253,080 (26.3%) | 48,700 (17.8%) |  |
|  |  |  |  |
| *Sector of working* |  |  |  |
| National Government | 24,540 (2.5%) | 7,610 (2.8%) | <0.001 |
| State/Territory/local government | 63,550 (6.6%) | 20,170 (7.4%) |  |
| Private sector | 520,930 (54.1%) | 177,270 (64.8%) |  |
| NA or missing | 354,700 (36.8%) | 68,710 (25.1%) |  |
|  |  |  |  |
| *Total personal income* |  |  |  |
| <= $64.999/year | 275,070 (28.5%) | 51,600 (18.8%) | <0.001 |
| $65.000-$103.999/year | 210,120 (21.8%) | 50,820 (18.6%) |  |
| >=104.000/year | 220,300 (22.9%) | 121,130 (44.2%) |  |
| NA or missing | 258,240 (26.8%) | 50,200 (18.3%) |  |
|  |  |  |  |
| *Hours worked in the week before Census* | 24.928 (22.529) | 31.453 (22.371) | <0·001 |
|  |  |  |  |
| *Long-term health condition(s)* |  |  |  |
| No long-term health condition | 481,920 (50.0%) | 160,390 (58.6%) | <0.001 |
| >=1 long-term health condition | 207,290 (21.5%) | 58,540 (21.4%) |  |
| NA or missing | 274,510 (28.5%) | 54,820 (20.0%) |  |
|  |  |  |  |
| **Household characteristics:** |  |  |  |
| *Family type* |  |  |  |
| Couple parent | 719,620 (74.7%) | 236,120 (86.3%) | <0.001 |
| Single parent | 244,100 (25.3%) | 37,640 (13.7%) |  |
|  |  |  |  |
| *Household size* | 4.161 (1.226) | 4.183 (1.237) | <0.001 |
|  |  |  |  |
| *Aboriginal & Torres Strait Islander family indicator* |  |  |  |
| Family with Aboriginal and/or Torres Strait Islander person(s) | 74,080 (7.7%) | 6,970 (2.5%) | <0.001 |
| Other families | 869,850 (90.3%) | 258,080 (94.3%) |  |
| NA or missing | 19,800 (2.1%) | 8,710 (3.2%) |  |
|  |  |  |  |
| **Geographical & socioeconomic characteristics of local area:** |  |  |  |
| *Remoteness* |  |  |  |
| Major Cities | 685,180 (71.1%) | 211,350 (77.2%) | <0.001 |
| Inner Regional | 176,740 (18.3%) | 46,950 (17.2%) |  |
| Outer Regional | 83,820 (8.7%) | 13,400 (4.9%) |  |
| Remote | 11,320 (1.2%) | 1,400 (0.5%) |  |
| Very Remote | 5,990 (0.6%) | 530 (0.2%) |  |
| NA or missing | 680 (0.1%) | 130 (0.0%) |  |
|  |  |  |  |
| *Socio-Economic Indexes for Area (SEIFA)* |  |  |  |
| 0-10% (least advantage) | 97,620 (10.1%) | 10,910 (4.0%) | <0.001 |
| 11-20% | 98,240 (10.2%) | 13,980 (5.1%) |  |
| 21-30% | 98,050 (10.2%) | 17,090 (6.2%) |  |
| 31-40% | 97,330 (10.1%) | 19,970 (7.3%) |  |
| 41-50% | 97,550 (10.1%) | 22,380 (8.2%) |  |
| 51-60% | 96,370 (10.0%) | 25,680 (9.4%) |  |
| 61-70% | 95,630 (9.9%) | 28,240 (10.3%) |  |
| 71-80% | 93,290 (9.7%) | 32,430 (11.8%) |  |
| 81-90% | 89,700 (9.3%) | 39,070 (14.3%) |  |
| 91-100% (most advantage) | 78,990 (8.2%) | 55,110 (20.1%) |  |
| NA or missing | 20,960 (2.2%) | 8,900 (3.3%) |  |

Notes: ^¶^ N (%) is for categorical variables; Mean (standard deviation) for continuous variables.

^Ŧ^ Test column reports P values from Pearson Chi-squared tests for categorical variables, and t tests for continuous variables.

Count numbers are rounded to nearest 10 per the ABS’s confidentiality rules.

**Supplementary Table 4: Contributions to influenza vaccination coverage gap between children attending public vs. private secondary schools in 2021 (robustness check)**

|  | **Model 1** | | **Model 2** | |
| --- | --- | --- | --- | --- |
|  | **Est. gap** **[relative contribution]** | **95% CI for est. gap** | **Est. gap [relative contribution]** | **95% CI for est. gap** |
| **Panel A. Influenza Vaccination Coverage rates** |  |  |  |  |
| Public schools | 0.139*** | (0.138; 0.139) | 0.147*** [0.0%] | (0.147; 0.148) |
| Private schools | 0.172*** | (0.170; 0.173) | 0.177*** [0.0%] | (0.175; 0.178) |
|  |  |  |  |  |
| **Panel B. Overall difference** | -0.033***[100.0%] | (-0.035; -0.032) | -0.029*** [100.0%] | (-0.031; -0.028) |
| Of which: |  |  |  |  |
| Explained | -0.011*** [33.2%] | (-0.012; -0.010) | -0.011*** [37.8%] | (-0.012; -0.010) |
| Unexplained | -0.022*** [66.8%] | (-0.024; -0.020) | -0.018*** [62.2%] | (-0.020; -0.016) |
|  |  |  |  |  |
| **Panel C. Explained part - detail** |  |  |  |  |
| *Child characteristics:* |  |  |  |  |
| Age | 0.000*** [-1.0%] | (0.000; 0.000) | 0.001*** [-2.2%] | (0.001; 0.001) |
| Gender | 0.000*** [0.4%] | (0.000; 0.000) | 0.000*** [0.4%] | (0.000; 0.000) |
| Aboriginal/Torres Strait Islander indicator | 0.001*** [-2.9%] | (0.001; 0.001) | 0.001*** [-1.9%] | (0.000; 0.001) |
| Citizenship status | -0.001*** [1.6%] | (-0.001; 0.000) | -0.001*** [2.4%] | (-0.001; -0.001) |
| Long-term health condition(s) | 0.001*** [-3.8%] | (0.001; 0.001) | 0.001*** [-2.8%] | (0.001; 0.001) |
| Additional language used at home | 0.001*** [-3.2%] | (0.001; 0.001) | 0.001*** [-4.5%] | (0.001; 0.001) |
|  |  |  |  |  |
| *Mother characteristics:* |  |  |  |  |
| Religion | -0.002*** [6.1%] | (-0.002; -0.002) | -0.001*** [5.1%] | (-0.002; -0.001) |
| English proficiency | 0.001*** [-2.7%] | (0.001; 0.001) | 0.001*** [-5.0%] | (0.001; 0.002) |
| Highest education obtained | -0.004*** [11.3%] | (-0.004; -0.003) | -0.002*** [8.1%] | (-0.003; -0.002) |
| Hours worked the week prior to census | 0.000*** [-1.0%] | (0.000; 0.000) | 0.000*** [-1.1%] | (0.000; 0.000) |
| Total personal income from all jobs | -0.001*** [2.2%] | (-0.001; 0.000) | 0.000 [1.0%] | (-0.001; 0.000) |
| Long-term health condition(s) | 0.001*** [-2.7%] | (0.001; 0.001) | 0.001*** [-2.1%] | (0.001; 0.001) |
|  |  |  |  |  |
| *Father characteristics:* |  |  |  |  |
| Highest education obtained |  |  | -0.004*** [13.7%] | (-0.004; -0.004) |
| Skill level of occupation |  |  | 0.000 [0.3%] | (-0.001; 0.000) |
| Hours worked the week prior to census |  |  | -0.001*** [2.6%] | (-0.001; -0.001) |
| Total personal income from all jobs |  |  | -0.002*** [8.3%] | (-0.003; -0.002) |
| Long-term health condition(s) |  |  | 0.001*** [-1.7%] | (0.000; 0.001) |
|  |  |  |  |  |
| *Family & household characteristics:* |  |  |  |  |
| Country of birth of parents | 0.000*** [-1.0%] | (0.000; 0.000) | 0.000*** [-0.8%] | (0.000; 0.000) |
| Family type | -0.004*** [11.0%] | (-0.004; -0.003) |  |  |
| Household size | 0.000*** [-0.5%] | (0.000; 0.000) | 0.000*** [1.6%] | (-0.001; 0.000) |
|  |  |  |  |  |
| *Geographical & socio-economics characteristics of local living area:* |  |  |  |  |
| Remoteness | 0.001*** [-1.8%] | (0.000; 0.001) | 0.001*** [-2.3%] | (0.001; 0.001) |
| SEIFA of local living area | -0.006*** [18.1%] | (-0.006; -0.006) | -0.004*** [14.9%] | (-0.005; -0.004) |
| State of residence | -0.001*** [3.2%] | (-0.001; -0.001) | -0.001*** [3.9%] | (-0.001; -0.001) |
|  |  |  |  |  |
| Total number of children | **1,191,490** | | **913,280** | |
| Of which: |  | |  | |
| Public schools | 886,240 | | 652,070 | |
| Private schools | 305,250 | | 261,210 | |

Notes: *** P value <0.01, ** P<0.05, and * P<0.1

Number of observations are rounded to the nearest 10 per the ABS’s confidentiality rules.

Estimated gaps and 95% CIs are rounded to three decimal places.

Model 1 controls for child, mother, family and local living area’s characteristics.

Model 2 adds to Model 1 father’s characteristics, therefore applied to children in couple parents’ families only.

The explained part (overall shown in Panel B; by each observed characteristics in Panel C) is the gap due to differences in observed characteristic. The unexplained part (Panel B) is the gap due to differences in estimated coefficients and other factors not captured in the model. Please see Supplementary Material 1 for details on the Oaxaca-Blinder decomposition method.

**Supplementary Figure 1: Influenza vaccination coverage by age-groups in 2021**

**Supplementary Figure 2: Influenza vaccination coverage in public vs. private secondary Schools by jurisdiction in 2021**

**References**

1. Oaxaca R. Male-Female Wage Differentials in Urban Labor Markets. *International economic review (Philadelphia)* 1973; **14**: 693-709.

2. Blinder AS. Wage Discrimination: Reduced Form and Structural Estimates. *The Journal of human resources* 1973; **8**: 436-55.

3. Australian Bureau of Statistics. Australian Standard Classification of Religious Groups. 2016.

4. Australian Bureau of Statistics. Australian Standard Classification of Languages. 2016.

5. Australian Bureau of Statistics. Australian Standard Classification of Cultural and Ethnic Groups. 2019.

6. Neumark D. Employers' Discriminatory Behavior and the Estimation of Wage Discrimination. *The Journal of human resources* 1988; **23**: 279-95.

7. Bauer TK, Sinning M. An extension of the Blinder–Oaxaca decomposition to nonlinear models. *Advances in statistical analysis : A journal of the German Statistical Society* 2008; **92**: 197-206.

8. Jann B. The Blinder–Oaxaca Decomposition for Linear Regression Models. *The Stata Journal* 2008; **8**: 453-79.

9. Yun M-S. Decomposing differences in the first moment. *Economics letters* 2004; **82**: 275-80.

10. Powers D, Yoshioka H, Yun M-S. MVDCMP: Stata module to compute multivariate decomposition for nonlinear response models. 2024.

11. Oaxaca RL, Ransom MR. On discrimination and the decomposition of wage differentials. *Journal of Econometrics* 1994; **61**: 5-21.

12. Fortin N, Lemieux T, Firpo S. Chapter 1 - Decomposition Methods in Economics. In: Ashenfelter O, Card D, editors. *Handbook of Labor Economics*: Elsevier; 2011. p. 1-102.

13. Karpf A, Mandel A. The changing value of the ‘green’ label on the US municipal bond market. *Nature Climate Change* 2018; **8**: 161-5.

14. Nguyen HT, Connelly LB, Le HT, Mitrou F, Taylor CL, Zubrick SR. Ethnicity differentials in academic achievements: the role of time investments. *Journal of Population Economics* 2020; **33**: 1381-418.

15. Bauer T, Göhlmann S, Sinning M. Gender differences in smoking behavior. *Health economics* 2007; **16**: 895-909.

16. Le H, Booth AL. Inequality in Vietnamese Urban–Rural Living Standards, 1993–2006. *Review of Income and Wealth* 2014; **60**: 862-86.

17. Nguyen HT, Brinkman S, Le HT, Zubrick SR, Mitrou F. Gender differences in time allocation contribute to differences in developmental outcomes in children and adolescents. *Economics of Education Review* 2022; **89**: 102270.
